# The Dichotomy of Mn–H Bond Cleavage and Kinetic Hydricity of Tricarbonyl Manganese Hydride Complexes

**DOI:** 10.3390/molecules28083368

**Published:** 2023-04-11

**Authors:** Elena S. Osipova, Sergey A. Kovalenko, Ekaterina S. Gulyaeva, Nikolay V. Kireev, Alexander A. Pavlov, Oleg A. Filippov, Anastasia A. Danshina, Dmitry A. Valyaev, Yves Canac, Elena S. Shubina, Natalia V. Belkova

**Affiliations:** 1 A.N. Nesmeyanov Institute of Organoelement Compounds, Russian Academy of Sciences (INEOS RAS), 28, Vavilova Str., 119334 Moscow, Russia; aosipova92@gmail.com (E.S.O.); kovalenko2000as@gmail.com (S.A.K.); elenor.kagami@gmail.com (E.S.G.); shu@ineos.ac.ru (E.S.S.); 2 LCC-CNRS, Université de Toulouse, CNRS, 205 Route de Narbonne, CEDEX 4, 31077 Toulouse, France; 3 Center of National Technological Initiative, Bauman Moscow State Technical University, 2nd Baumanskaya Str., 5, 105005 Moscow, Russia; 4 Moscow Institute of Physics and Technology, Institutskiy per., 9, 141700 Dolgoprudny, Russia

**Keywords:** manganese hydrides, hydrogen bond, non-covalent interactions, proton transfer, hydride transfer, hydricity

## Abstract

Acid-base characteristics (acidity, p*K*a, and hydricity, ΔG°_H−_ or *k*_H−_) of metal hydride complexes could be a helpful value for forecasting their activity in various catalytic reactions. Polarity of the M–H bond may change radically at the stage of formation of a non-covalent adduct with an acidic/basic partner. This stage is responsible for subsequent hydrogen ion (hydride or proton) transfer. Here, the reaction of tricarbonyl manganese hydrides *mer*,*trans*–[L_2_Mn(CO)_3_H] (**1**; L = P(OPh)_3_, **2**; L = PPh_3_) and *fac*–[(L–L′)Mn(CO)_3_H] (**3**, L–L′ = Ph_2_PCH_2_PPh_2_ (dppm); **4**, L–L′ = Ph_2_PCH_2_–NHC) with organic bases and Lewis acid (B(C_6_F_5_)_3_) was explored by spectroscopic (IR, NMR) methods to find the conditions for the Mn–H bond repolarization. Complex **1**, bearing phosphite ligands, features acidic properties (p*K*a 21.3) but can serve also as a hydride donor (ΔG^≠^_298K_ = 19.8 kcal/mol). Complex **3** with pronounced hydride character can be deprotonated with KHMDS at the CH_2_–bridge position in THF and at the Mn–H position in MeCN. The kinetic hydricity of manganese complexes **1**–**4** increases in the order *mer*,*trans*–[(P(OPh)_3_)_2_Mn(CO)_3_H] (**1**) < *mer*,*trans*–[(PPh_3_)_2_Mn(CO)_3_H] (**2**) ≈ *fac*–[(dppm)Mn(CO)_3_H] (**3**) < *fac*–[(Ph_2_PCH_2_NHC)Mn(CO)_3_H] (**4**), corresponding to the gain of the phosphorus ligand electron-donor properties.

## 1. Introduction

A search for inexpensive and efficient catalysts that give more sustainable alternatives to platinum group metals recently led to remarkable progress in the development of catalytic systems based on organometallic manganese complexes [1,2]. While pincer-type Mn(I) derivatives still dominate in the field of catalytic (de)hydrogenation, it was demonstrated that less elaborated bidentate systems *fac*–[(L–L′)Mn(CO)_3_Br] may also be highly efficient [3]. Generally, the formation of catalytically relevant transition metal hydrides from the corresponding bromide precursors proceeds in situ in the presence of various basic additives. This activation step can be nicely illustrated for Mn(I) complexes *fac*–[(P–NHC)Mn(CO)_3_Br] (P–NHC = *κ*^2^*P*,*C*–Ph_2_PCH_2_–NHC) [4] and *fac*–[(dppm^R^)Mn(CO)_3_Br] (dppm = *κ*^2^*P*,*P*–Ph_2_PCH(R)PPh_2_, R = H, Me, Ph) [5] in the presence of KHMDS leading to the formation of cyclometalated species capable of activating dihydrogen via unconventional metal-ligand cooperation. The resulting hydride products *fac*–[(L–L′)Mn(CO)_3_H] interact with organic substrates to form non-covalent adducts and typically the formation of such intermediates directly precedes hydride/proton transfer steps providing *in fine* the hydrogenation of polar C=X bonds.

**Scheme 1:**
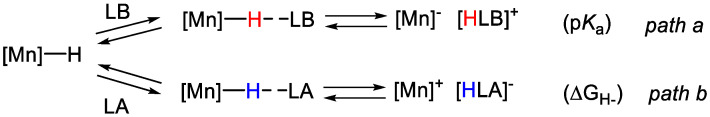
LB = Lewis base, LA = Lewis acid.

It is generally accepted that transition metal hydrides can be proton, hydride or hydrogen atom donors [6,7,8]. However, the vast majority of hydride complexes exhibit only one reactivity mode being determined by the nature of auxiliary ligands and relative charge on the core metal atom [9]. Nevertheless, there are a few families of hydrides in which the same hydride complex possesses distinct reactivity. *Dual* reactivity is well documented, for example, for groups 6–8 metal complexes [CpM(CO)_3_H] (M = Mo, W), [(CO)_5_MH] (M = Mn, Re) and [CpM(CO)_2_H] (M = Fe, Ru, Os) [8,10,11,12]. Whether the M–H bond releases a proton or hydride, obviously, depends on the partner reagent. We have shown for the first time that the M–H bond polarity gets adjusted at the stage of a non-covalent complex formation preceding the M–H bond dissociation [13] (Scheme 1).

The acidity and hydricity of the M–H bond can be quantified in terms of p*K*_a_ and ΔG°_H−_, respectively [14,15,16]. The quantitative scale of kinetic hydricity (*k*_H−_) has been developed for group 6–7 metal hydrides in the pioneering work of Bullock [11]. However, the data for manganese complexes are still scarce despite their utility as potential catalysts. Experimental values of acidity and hydricity are known only for [(CO)_5_MnH] (p*K*_a_ = 14.2 in MeCN [8], ΔG°_H−_ = 59.61 kcal/mol [17], *k*_H−_ = 50 M^−1^s^−1^ [11]), [(CO)_4_(C_6_H_6_)MnH] (p*K*_a_ = 26.8 in MeCN [8]) and *cis*–[(CO)_4_(PPh_3_)MnH] (p*K*_a_ = 20.4 in MeCN [8], *k*_H−_ = 230 M^−1^s^−1^ [11]). There is a sole theoretical work devoted to a systematic study of (CO)_5_MnH and (CO)_5-n_(PH_3_)_n_MnH complexes [17] demonstrating that the hydricity of the metal hydride bond is greatly amplified when the CO ligand is replaced by a phosphine donor [17].

The research presented herein explores the dichotomy of Mn–H bond cleavage and the potential of its repolarization entailed by intermolecular interactions with Lewis acids and bases. By gaining an understanding of the acid-base characteristics of different manganese hydrides, it should be possible to forecast their catalytic performance and drive the catalyst design.

**Scheme 2:**
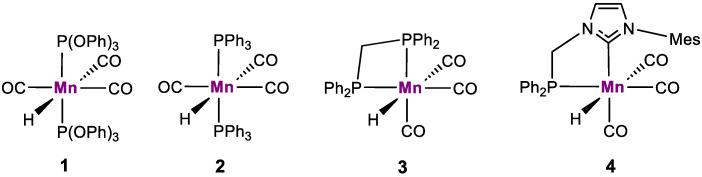
Representation of the Mn–H complexes **1**–**4** which are the subject of this study.

In this context, we chose two types of octahedral Mn(I) hydride complexes (Scheme 2) in which the Mn–H bond is expected to have different polarity. Electron-deficient phosphite ligands in complex *mer*,*trans*–[(P(OPh)_3_)_2_Mn(CO)_3_H] (**1**) and more donating triarylphosphines in complex *mer*,*trans*–[(PPh_3_)_2_Mn(CO)_3_H] (**2**) should provide them an acidic and basic character, respectively. In agreement with our expectations, complex **1** can be deprotonated by *t*BuOK [18], and complex **2** exhibits typical reactivity of “hydridic” hydride releasing H_2_ [19,20] upon protonation by HBF_4_·Et_2_O [21]. The incorporation of chelating and more electron-rich ligands such as bidentate phosphine in *fac*–[(dppm)Mn(CO)_3_H] (**3**) or phosphine–N–Heterocyclic carbene in *fac*–[(P–NHC)Mn(CO)_3_H] (**4**) ought to increase the Mn–H basicity and hydride donating ability (Scheme 2). The acidity and hydricity of **1**–**4** have not been evaluated before this work; therefore, the entire set of these complexes could be used to create the scale of their thermodynamic/kinetic hydricity by evaluating the impact of the ligand.

## 2. Results and Discussion

### 2.1. Interaction of Tricarbonyl Manganese Hydrides ***1***–***4*** with Bases

*Hydrogen bonding.* Addition of Lewis bases (LB) such as pyridine and hexamethylphosphoramide (HMPA) to complex *mer*,*trans*–[(P(OPh)_3_)_2_Mn(CO)_3_H] (**1**) in methylcyclohexane (MCH) at 190 K leads to weak hydrogen bond formation where Mn–H serves as a proton donor [22,23,24,25]. Hydrogen bonding was evidenced by the appearance of a low-frequency shoulder at the initial ν_CO_ 1958 cm^−1^ band (Δν 5–20 cm^−1^) (Figure S1). Since hydrogen bonding is a reversible process, a temperature increase up to 290 K shifts the equilibrium (Scheme 1, path a) towards free manganese hydride. Complex *fac*–[(dppm)Mn(CO)_3_H] (**3**) also forms a hydrogen bond with these bases; its three ν_CO_ bands (1996, 1916, 1909 cm^−1^) shift to lower frequencies by 3–7 cm^−1^ in the presence of 70 equiv. HMPA (Figure S2). However, the strength of these bases is insufficient for proton transfer to occur. The change of the nonpolar MCH to polar acetonitrile also does not promote the proton transfer from **3** to pyridine (p*K*_a_ = 12.53 in MeCN) or HMPA (p*K*_a_ = 6.1 in CH_3_NO_2_).

*Proton transfer.* Quantitative deprotonation of **1** was observed in acetonitrile when a stronger base—1,8–diazabicyclo[5.4.0]undec–7–ene (DBU; p*K*_a_ = 24.31 in MeCN)—was used. Full conversion of **1** to anionic complex [(P(OPh)_3_)_2_Mn(CO)_3_]^−^[HDBU]^+^ (**1^−^**) was confirmed by ^31^P NMR in CD_3_CN, since the initial signal at δ_P_ 183.3 ppm converts into downfield resonance δ_P_ 206.5 ppm after DBU addition (the key spectral parameters for this and all other complexes studied are summarized in Table 1). To explore the features of proton transfer equilibrium (Scheme 1, path a), we decided to return into a non-polar solvent that should impede proton transfer process. Indeed, in methylcyclohexane the proton transfer is temperature dependent and starts when the reaction mixture is cooled below 260 K. According to IR spectra, the addition of 1.1 equiv. DBU to a solution of **1** in MCH at low temperatures (190–260 K) leads to the appearance of anionic manganese species [(P(OPh)_3_)_2_Mn(CO)_3_]^−^ (**1^−^**, ν_CO_ 1815 cm^−1^) at the expense of the initial complex (ν_CO_ 1958 cm^−1^). Unexpectedly, proton transfer is slow at these temperatures, taking 1.2 h at 230 K to reach the equilibrium (Figure S3). The rate constants analysis gave the activation energies (See Supplementary Materials for more details) for the proton transfer step: Δ*H*^≠^ = 7.5 kcal/mol, Δ*S*^≠^ = −26 cal/(mol·K), Δ*G*^≠^_298K_ = 15.3 kcal/mol. Since the system does come to equilibrium, the experimental equilibrium constants with thermodynamic parameters of proton transfer in MCH were obtained: Δ*H*ᵒ = −13.1 kcal/mol, Δ*S*ᵒ = −50 cal/(mol·K), Δ*G*ᵒ_298K_ = 1.9 kcal/mol (See Supplementary Materials for more details). The reaction of **1** with a slightly weaker base [Bu_4_N]^+^[4–NO_2_C_6_H_4_O]^−^ (p*K*_a_ = 20.7 in MeCN [26]) immediately reaches equilibrium in MeCN with the formation of only ~30% proton transfer product (Figure S4). The known acidity constant of 4–NO_2_C_6_H_4_OH allows an estimation of complex **1** acidity p*K*_a_(**1**) = 21.3.

Manganese catalyzed transfer hydrogenation reactions are usually carried out in the presence of strong bases such as potassium *tert*-butylate or KHMDS [1,3,4]. A possible reaction mechanism includes Mn–H bond deprotonation with formation of anionic complex, which participates in further catalytic transformations [3]. We have tried multiple times to identify the conditions for proton abstraction from *mer*,*trans*–[(PPh_3_)_2_Mn(CO)_3_H] (**2**) and *fac*–[(dppm)Mn(CO)_3_H] (**3**), where the Mn–H bond is expected to be rather electron-rich due to the presence of phosphine ligands. However, no repolarization of the Mn–H bond and proton transfer was observed for hydride complexes **2** and **3** with various bases (pyridine, HMPA, DBU, TBD (1,5,7–triazabicyclo[4.4.0]dec–5–ene, p*K*_a_ = 26.03 in MeCN [28])) even in polar solvents (MeCN, THF). Fortunately, treatment of complex **2** at ambient temperature in THF with KHMDS excess (2 equiv., p*K*_a_ = 26 in THF [29]) led to partial proton transfer indicated by the appearance of two new low-frequency ν_CO_ bands at 1770 and 1741 cm^−1^ in IR spectrum that correspond to anionic complex **2^−^** (Figure S5) [30]. From these spectral data the p*K*_a_ value 27.3 for manganese hydride **2** in THF was estimated.

**Scheme 3:**
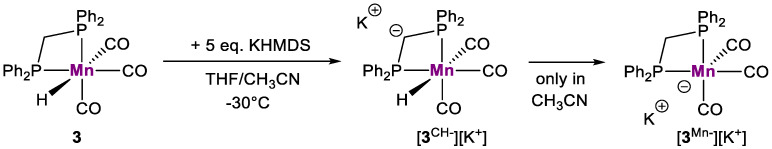
Formation of [**3**^Mn−^][K^+^] from **3** by addition of KHMDS.

**Figure 1:**
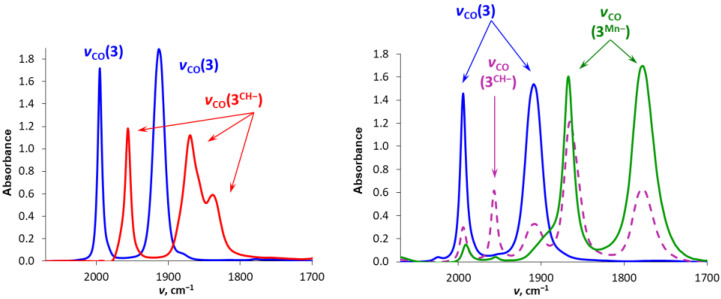
IR spectra of *fac*–[(dppm)Mn(CO)_3_H] (**3**) (*c* = 0.01 M) alone (blue) and after KHMDS addition (5 equiv.; red/green). (**Left**)—THF, (**right**)—MeCN (*l* = 0.01 cm, 295 K).

The reaction of hydride complex **3** with KHMDS (5 equiv.) in THF leads to full proton transfer and the appearance of three new low-frequency-shifted CO bands (1957, 1871, 1876 cm^−1^) in the IR spectra (Figure 1). The shift of ν_CO_ bands by ca. 40 cm^−1^ in the IR spectra is not consistent with the presence of the negative charge at the metal atom in the case of Mn–H bond deprotonation. Moreover, ^1^H NMR spectrum shows the presence of a triplet signal at δ_H_ −5.54 ppm in the hydride region, while for the initial complex **3** the hydride signal (δ_H_ −5.53 ppm) is a triplet of doublets due to the ^2^*J*_PH_ and an additional ^4^*J*_HH_ spin-spin interaction with one of the CH_2_ bridge H-atoms. The phosphorus resonance observed in this **3**/KHMDS mixture by ^31^P{^1^H} NMR spectroscopy is shifted to stronger field (δ_P_ 10.9) relative to that (δ_P_ 30.1) of the initial manganese hydride **3** (Figure S6). These spectral data allow the proposal that deprotonation occurs at the bridging CH_2_ group of dppm ligand with the formation of [**3**^CH−^][K^+^]. In the ^13^C spectrum, the triplet (δ_C_ 20.8, t, ^1^*J*_CP_ = 51.4 Hz, PC*H*P) of corresponding anionic carbon CH^−^ is also shifted to stronger field compared to the CH_2_ signal of **3** (δ_C_ 48.0, t, ^1^*J*_CP_ = 22.4 Hz, PC*H*_2_P). The estimated p*K*_a_ value of methylene CH-proton in complex **3** equals 26 in THF. A more polar acetonitrile deprotonation of **3** by the same amount of KHMDS goes through [**3**^CH−^][K^+^] formation yielding ultimately the anionic manganese complex [**3**^Mn−^][K^+^] (Scheme 3). In the IR and NMR spectra, measured immediately after mixing, three manganese species could be observed; however, in 15 min, the intermediate [**3**^CH−^][K^+^] (ν_CO_ 1956, 1870 cm^−1^; δ_P_ 10.4 ppm) completely transforms into the final species [**3**^Mn−^][K^+^] ((ν_CO_ 1867, 1779 cm^−1^; δ_P_ 29.9 ppm) (Figure 1 and Figure S6) that has no hydride signal in the ^1^H NMR spectrum. According to our DFT calculations, the intramolecular proton transfer from manganese to the anionic bridge CH^−^ is explained by thermodynamic preference of the metal deprotonated form (ΔG_298K_ = −0.2 kcal/mol) in acetonitrile compared with THF (Table 2). That implies that even manganese complexes with pronounced hydride character [11,17] can be deprotonated by KHMDS, thus allowing the corresponding anionic intermediates to participate in hydrogenation reactions.

### 2.2. Interaction of Tricarbonyl Manganese Hydrides with Lewis Acids

**Scheme 4:**
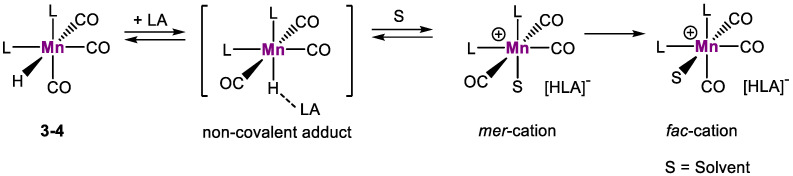
Isomerization process of complexes **3**–**4** into more stable *facial* isomers.

*Non-covalent adducts.* Interaction of manganese hydride complexes (**1**–**4**) with Lewis acids (**LA**) leads to Mn–H bond polarization, making the hydride atom more negatively charged. Recently, we have proven that the formation of non-covalent adducts between Lewis acid and hydride complex *fac*–[(dppm)Mn(CO)_3_H] (**3**) at 180 K precedes the hydride ion abstraction [27] (Scheme 4). Noteworthy, while the initial hydride **3** has *facial* geometry, the most stable form of the non-covalent adduct is the isomer with the *meridional* geometry. The latter then undergoes additional transformations, including intramolecular hydride transfer with the formation of *meridional* cation stabilized by the solvent molecule *mer*–[(dppm)Mn(CO)_3_(κ^1^*Cl*–CH_2_Cl_2_)](HBAr_3_) and its isomerization to a more stable *facial* isomer *fac*–[(dppm)Mn(CO)_3_(κ^1^*Cl*–CH_2_Cl_2_)](HBAr_3_).

**Figure 2:**
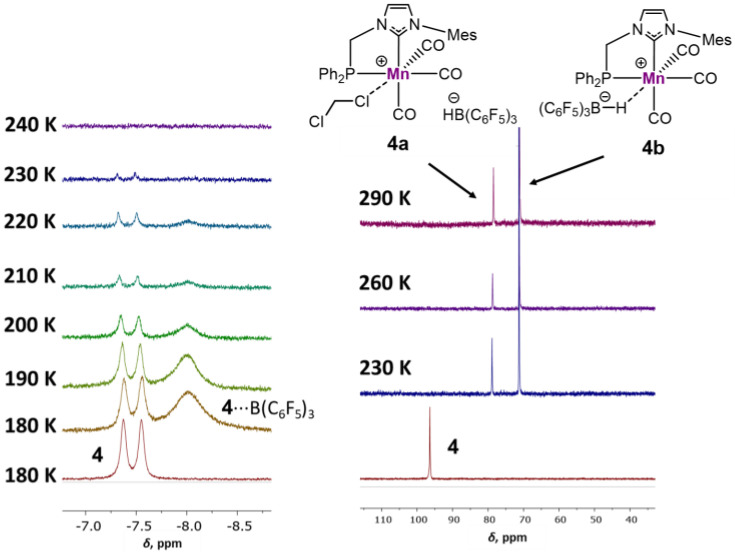
Variable temperature ^1^H (300 MHz) and ^31^P{^1^H} (162.0 MHz) NMR spectra of complex **4** in CD_2_Cl_2_ solution (bottom lines) and its mixture with B(C_6_F_5_)_3_.

The analogous chemical behavior was observed upon addition of B(C_6_F_5_)_3_ to complex *fac*–[(P–NHC)Mn(CO)_3_H] (**4**) at 180 K in methylene chloride. The Lewis acid coordination to the hydride ligand and formation of the non-covalent adduct **4**···LA gives in ^1^H NMR spectrum a broadened resonance at δ_H_ −7.98 ppm shifted high-field relative to the initial signal of **4** (δ_H_ −7.42) (Figure 2). A weak BrØnsted acid *mer*,*trans*–[(P(OPh)_3_)_2_Mn(CO)_3_H] (**1**) also interacts with Lewis acids as a hydride-ion donor. Addition of B(C_6_F_5_)_3_ (10 equiv.) to the toluene solution of **1** (ν_CO_ 2040 w, 2028 w, 1956 s cm^−1^) at 190 K leads to the formation of a non-covalent complex **1**···B(C_6_F_5_)_3_ (ν_CO_ 2035 s, 1973 s cm^−1^). We assume that this adduct has a *facial* configuration, since its ν_CO_ bands are strongly shifted to high frequencies (*cf*. 2046 s, 1980 s cm^−1^ for *fac*-**1** and 2026 w, 1942 s cm^−1^ for *mer*,*trans*-**1** in nujol) [31]. Formation of non-covalent adduct with B(C_6_F_5_)_3_ stabilizes *fac*-**1** and in the 190–220 K temperature range the equilibrium between two species, *mer*,*trans*-**1** and *fac*-**1**···B(C_6_F_5_)_3_, is observed in the IR spectra (Figure S7). In ^1^H NMR spectra of the **1** + B(C_6_F_5_)_3_ mixture, measured under the same conditions, the formation of the intermediate complex *fac*-**1**⋯B(C_6_F_5_)_3_ is evidenced by new broad hydride resonance shifted to the higher field (δ_H_ −9.36 ppm) (Figure S8, left). In ^31^P{^1^H} NMR spectra (Figure S8, right), the resonance at δ_P_ 163 ppm exhibits the same temperature behavior and can be attributed to *fac*-**1**⋯B(C_6_F_5_)_3_. Thus, complex **1** is the least reactive toward hydride transfer (see below) and its non-covalent adduct with Lewis acid exists in a wider temperature range.

*Hydride transfer.* The non-covalent adducts **1**⋯LA–**4**⋯LA precede the intramolecular hydride transfer that yields the cationic complexes with different geometries, which are stabilized by coordination of solvent molecule or [H-LA]^−^ anion. Thus, for *fac*–[(P–NHC)Mn(CO)_3_H] (**4**) interacting with B(C_6_F_5_)_3_ at 180 K, new high-frequency ν_CO_ bands at 2038, 1968 and 1942 cm^−1^ appear in the IR spectrum assigned to the cationic *mer*-intermediate; at 190 K they already transform into *fac*-product with ν_CO_ bands at 2032, 1949 and 1921 cm^−1^ (Figure S9). In contrast to isostructural *fac*–[(dppm)Mn(CO)_3_H] (**3**), in the case of complex **4** two configurations are possible when Mn(CO)_3_ moiety is in meridional arrangement: hydride ligand being *trans* to the phosphorus atom (*mer*-P-**4**) or *trans* to the NHC carbon atom (*mer*-C-**4**) (Figure S8). Since complex *mer*-P-**4** should feature more hydridic H-atom than *mer*-C-**4** due to the higher *trans*-effect of phosphorus [32], we assumed that the hydride transfer occurs through the formation of the meridional *trans*-to-P form (*mer*-P-**4**⋯B(C_6_F_5_)_3_).

**Figure 3:**
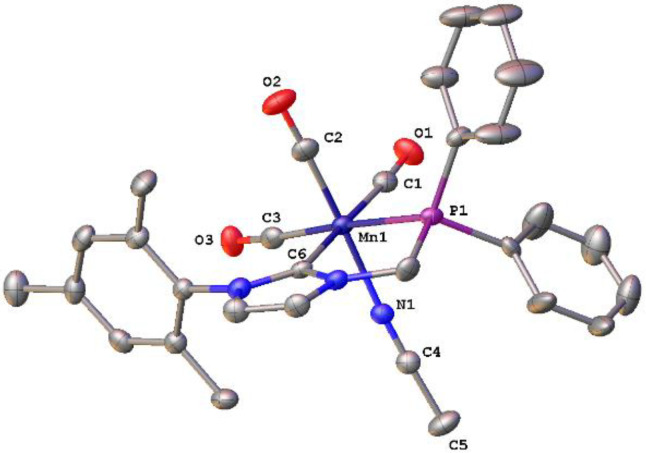
Molecular structure of the cationic complex *fac*–[(P–NHC)Mn(CO)_3_(MeCN)]^+^[BF_4_]^−^ (**4^MeCN^**) (40% probability ellipsoids, only one crystallographically independent molecule in the cell is shown). Counter-anions and hydrogen atoms are omitted for clarity. Selected bond lengths (Å): Mn1-N1 2.011(4), Mn1-P1 2.317(1), Mn1-C6 2.048(5), C1-O1 1.146(7), C2-O2 1.156(6), C3-O3 1.146(5), Mn1-C1 1.817(6), Mn1-C2 1.786(5), Mn1-C3 1.835(4), N1-C4 1.130(6).

Above 230 K, the equilibrium is completely shifted to cationic products, the ^31^P{^1^H} spectrum contains only two resonances δ_P_ 78.0 and 71.0 ppm in 1:4 ratio (Figure 2). After 4 days at room temperature, this ratio inverts and becomes 2:1. By selectively decoupling ^31^P, the CH_2_-bridge H-atoms δ_H_ 5.07 (dd, ^2^*J*_HH_ = 14.0, ^2^*J*_PH_ = 5.1 Hz) and 4.94 (dd, ^2^*J*_HH_ = 14.1, ^2^*J*_PH_ = 6.6 Hz) can be attributed to complex **4a** with δ_P_ 78.1 ppm and δ_H_ 5.48 (vt, ^2^*J*_HH_ = 14.4 Hz, ^2^*J*_PH_ = 13.7 Hz) and 4.99 (d, ^2^*J*_HH_ = 14.4 Hz) to complex **4b** δ_P_ 71.1. While the ^31^P{^1^H} resonance of **4a** shifts (between 74.1 and 78.1 ppm) in response to changing the solvent (toluene, *n*BuCl, C_6_H_5_Cl, CD_2_Cl_2_), the chemical shift of **4b** keeps its position (Table S1). Thus, we suggest that the two cationic products are [(P–NHC)Mn(CO)_3_(Solvent)][HB(C_6_F_5_)_3_)] (**4a**), which has a coordinated solvent molecule, and [(P–NHC)Mn(CO)_3_][HB(C_6_F_5_)_3_)] (**4b**), which is a contact ionic pair stabilized by B–H bond interaction with the cationic metal center (**4b**) remains in tranquility. The sensitivity of the ^31^P{^1^H} resonance of **4a** to the media is indicative of the solvent’s influence on its structure. To obtain an independent proof of its structure, we reacted the manganese bromide *fac*–[(P–NHC)Mn(CO)_3_Br] with AgBF_4_ in MeCN as a solvent. The cationic complex **4^MeCN^** obtained features acetonitrile molecule coordinated to the metal center as it was confirmed by X-ray diffraction (Figure 3), and its ^31^P{^1^H} resonance (77.5 ppm in CD_2_Cl_2_) is in the range of **4a**-type complexes.

**Scheme 5:**
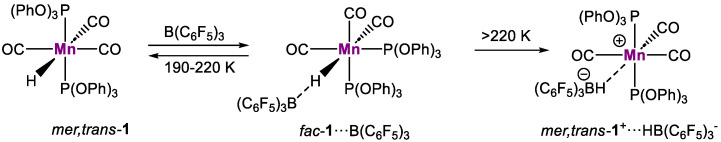
Formation of complex *mer*,*trans*–[1]^+^[HB(C_6_F_5_)_3_] from **1** in the presence of B(C_6_F_5_)_3_.

For the non-covalent complex *fac*-**1**⋯B(C_6_F_5_)_3_ the hydride transfer occurs above 230 K (Scheme 5). The single product of the hydride transfer is a cationic manganese complex *mer*,*trans*–[1]^+^[HB(C_6_F_5_)_3_]. The signal of the cationic product [1]^+^[HB(C_6_F_5_)_3_]^−^ becomes clearly seen in ^31^P spectra at 250 K (δ_P_ 153.0 ppm).

**Figure 4:**
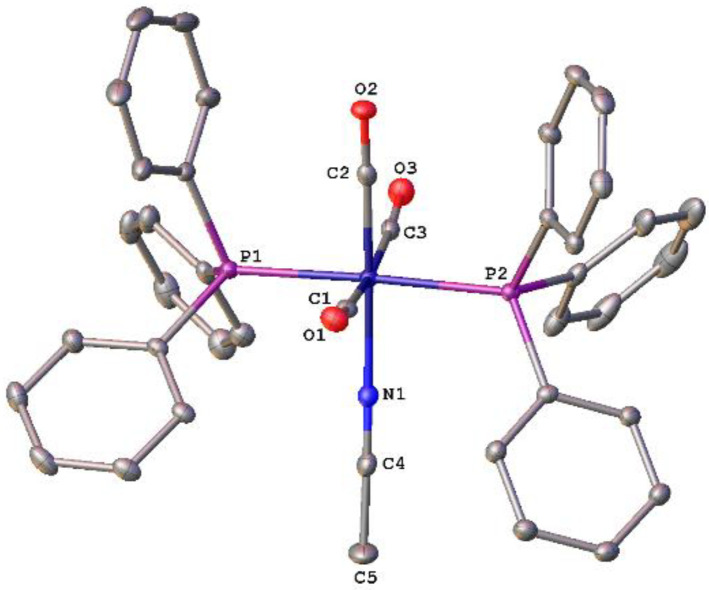
Molecular structure of the cationic complex *mer*,*trans*–[(PPh_3_)_2_Mn(CO)_3_(CH_3_CN)]^+^ **2^MeCN^** (40% probability ellipsoids, tetrafluoroborate anion, solvate molecule of dichloromethane and hydrogen atoms are omitted for clarity). Selected bond lengths (Å): Mn1-N1 1.999(3), Mn1-P1 2.3337(8), Mn1-P2 2.3329(8), C1-O1 1.140(3), C2-O2 1.149(4), C3-O3 1.137(3), Mn1-C1 1.857(3), Mn1-C2 1.794(3), Mn1-C3 1.860(4), N1-C4 1.134(4).

Structurally similar complex *mer*,*trans*–[(PPh_3_)_2_Mn(CO)_3_H] (**2**) bearing more donating phosphine ligands was found to be more reactive. Indeed, it transforms into *mer*,*trans*–[(PPh_3_)_2_Mn(CO)_3_(solv)][HB(C_6_F_5_)_3_] (**2**^+^) already at 180 K (Figure S11). IR spectra in the 180–250 K range show the transformation of hydride **2** directly into cationic complex [**2^+^**][HB(C_6_F_5_)_3_] (ν_CO_ 1980, 1930 cm^−1^) without detectable non-covalent adduct formation and *mer*-to-*fac* isomerization. At temperatures higher than 250 K, partial decomposition of this cationic species was observed, resulting in the formation of known tetracarbonyl complex *trans*–[(PPh_3_)_2_Mn(CO)_4_]^+^ exhibiting a ν_CO_ band at 2002 cm^−1^ [33]. ^31^P{^1^H} NMR spectra confirm the formation of sole cationic product (δ_P_ 61.8 ppm) and its further decomposition. Direct synthesis of *mer*,*trans*–[(PPh_3_)_2_Mn(CO)_3_(CH_3_CN)]^+^[BF_4_]^−^ **2^MeCN^** allows the formation of a stable cationic product with ν_CO_ at 2055 w, 1977 s, 1946 s cm^−1^ characterized by X-ray diffraction (Figure 4).

### 2.3. Kinetic Hydricity of Manganese Tricarbonyl Complexes

The hydride transfer from complexes **1**–**4** to B(C_6_F_5_)_3_ is relatively slow at low temperatures that allow the estimation of their kinetic hydricities (See Supplementary Materials for more details; Figures S12 and S13). The low-temperature IR monitoring of hydride transfer kinetics in the presence of 1.1 equiv. B(C_6_F_5_)_3_ in *n*BuCl gave effective rate constants (*k_eff_*). The value of free energy ΔG^≠^_298K_ characterizes the kinetic hydricity of manganese complexes but depends on the Lewis acidity of the given hydride abstractor. As expected, the hydride complex **1**, which is a weak acid due to electron-withdrawing phosphite ligands, has the lowest hydricity (ΔG^≠^_298K_ = 19.8 kcal/mol), while complex **4** bearing phosphine-carbene ligand, the most electron-rich in this series, possesses the highest hydricity (ΔG^≠^_298K_ = 16.5 kcal/mol) among the complexes studied (Table 3). The kinetic hydricity of the manganese complexes of interest increases in the order *mer*,*trans*–[(P(OPh)_3_)_2_Mn(CO)_3_H] (**1**) < *mer*,*trans*–[(PPh_3_)_2_Mn(CO)_3_H] (**2**) ≈ *fac*–[(dppm)Mn(CO)_3_H] (**3**) < *fac*–[(P-NHC)Mn(CO)_3_H] (**4**), reflecting the gain of the phosphorus ligand electron donor properties (basicity). On the activation free energy scale (Δ*G*^≠^_298K_) there is only small by 0.5 kcal/mol difference between complexes **2**–**4** bearing electron-donating ligands. However, the distinction between their properties becomes more pronounced at lower temperatures due to the impact of highly negative activation entropy ∆S^≠^ (cf. *k_eff_*_220K_, Table 3).

## 3. Materials and Methods 

All reactions were performed using standard Schlenk procedures under a dry argon atmosphere. Dry and oxygen-free organic solvents (toluene, THF) were obtained using a solvent purification system from M. Braun (Garching, Germany). Methylcyclohexane was dried over Na and distilled under an argon atmosphere. *n*BuCl, CH_2_Cl_2_ and acetonitrile were stored over CaH_2_ and distilled under an argon atmosphere before use. Deuterated solvents for NMR were degassed before use by three freeze–pump–thaw cycles and kept over 3Å molecular sieves. A liquid nitrogen/ethanol or nitrogen/isopropanol slush bath was used to maintain samples at the desired low temperature.

Variable-temperature (VT) NMR spectra were recorded on Bruker Avance 300, Bruker Avance 400 (Bruker, Billerica, MA, USA) and Varian Inova 400 (Varian, Palo Alto, CA, USA) spectrometers operating at 300 and 400 MHz in the 180–300 K temperature range. ^1^H and ^13^C{^1^H}, chemical shifts are reported in parts per million (ppm) downfield of tetramethylsilane (TMS) and were calibrated against the residual resonance of the deuterated solvent, while ^31^P{^1^H} chemical shifts were referenced to 85% H_3_PO_4_ with downfield shift taken as positive. The IR spectra were recorded at different temperatures (160–293 K) using a home-modified cryostat (Carl Zeiss Jena) with a Nicolet iS50 FTIR (Thermo Scientific, Waltham, MA, USA) spectrometer using 0.05 cm CaF_2_ cells. The accuracy of the experimental temperature adjustment was ±0.5 °C. The cryostat modification allowed the transfer of the reagents (premixed at either low or room temperature) under an inert atmosphere directly into the cells.

Manganese hydride complexes *mer*,*trans*–[(P(OPh)_3_)_2_Mn(CO)_3_H] (**1**) [31], *mer*,*trans*–[(PPh_3_)_2_Mn(CO)_3_H] (**2**) [34], *fac*–[(dppm)Mn(CO)_3_H] (**3**) [27] and *fac*–[(P-NHC)Mn(CO)_3_H] (**4**) [4] (Scheme 2) were prepared according to the literature methods. Commercially available tris(pentafluorophenyl)boron was purified by sublimation before use. All other reagent-grade chemicals purchased from commercial sources were used as received.

Synthesis of fac–[(P–NHC)Mn(CO)_3_(MeCN)]BF_4_ (4^MeCN^)

AgBF_4_ (0.375 mmol, 73 mg) was placed into the Schlenk flask containing *fac*-(P-NHC)Mn(CO)_3_Br (0.375 mmol, 226 mg) under inert atmosphere. Then 5 mL of CH_3_CN was added upon stirring at room temperature. The obtained suspension was sonicated for 5 min and left stirring overnight till complete product formation that was controlled by the IR spectroscopy. After that, the CH_3_CN was removed under reduced pressure, the yellow oil residue was dissolved in 5 mL of CH_3_CN again to avoid colloid solution formation. The resulting solution was separated from the precipitate via filtration through a Pasteur pipette with Celite, concentrated to 1/10 of initial volume, and dry Et_2_0 (80 mL) was added dropwise to induce precipitation of the product. The precipitate was left under supernatant overnight at −20 °C. The next day, the supernatant was removed, and the precipitate was washed with 5 mL two times and dried in a vacuum yielding (211 mg, 86%) pale-yellow powder.

Synthesis of mer,trans–[(PPh_3_)_2_Mn(CO)_3_(CH_3_CN)][BF_4_] (2^MeCN^)

A suspension of (PPh_3_)_2_Mn(CO)_3_H (0.008 mmol, 5.0 mg) and [Ph_3_C][BF_4_] (0.008 mmol, 2.6 mg) in CH_3_CN (5 mL) was placed in the ultrasonic bath for 5 min at room temperature and left stirring till complete product formation that was controlled by the IR spectroscopy. The solvent was removed under reduced pressure. The resulting pale-yellow solid was dissolved in CH_2_Cl_2_ (1 mL) and then filtered through a Pasteur pipette with Celite. Hexane (5 mL) was added to the solution to induce crystallization. The supernatant was removed by decantation, the crystalline precipitate was washed with hexane (2 × 5 mL) and then dried in the vacuum to yield [(PPh_3_)_2_Mn(CO)_3_(CH_3_CN)][BF_4_] (4.6 mg, 76%) as pale-yellow crystals.

Structures of reactants and complexes were optimized at the ωB97-XD level [35], applying def2-TZVP basis set [36] by Gaussian 09 [37]. Optimizations were done in toluene, THF and CH_3_CN introduced by a SMD solvent model [38].

Cationic complexes *mer*,*trans*–[(PPh_3_)_2_Mn(CO)_3_(MeCN)][BF_4_] **2^MeCN^** and *fac*–[(P–NHC)Mn(CO)_3_(MeCN)][BF_4_] **4^MeCN^** were crystalized from the CH_2_Cl_2_/hexane system. X-ray diffraction data were collected at 100 K with a Bruker Quest D8 CMOS diffractometer (Bruker, MA, USA), using graphite monochromated Mo-Kα radiation (λ = 0.71073 Å). Using Olex2 [39], the structures were solved with the ShelXT [40] structure solution program using Intrinsic Phasing and refined with the XL [41] refinement package using Least-Squares minimization against F^2^ in anisotropic approximation for non-hydrogen atoms. Positions of hydrogen atoms were calculated, and they were refined in the isotropic approximation within the riding model. Crystal data and structure refinement parameters are given in Table S2. CCDC 2241621 and 2241622 contain the supplementary crystallographic data for **2^MeCN^** and **4^MeCN^**, respectively.

### 3.1. General Procedure for the Interaction with Bases

For Variable Temperature IR Studies

The solution of *mer*,*trans*–[(P(OPh)_3_)_2_Mn(CO)_3_H] (**1**, c = 0.003 M) was prepared at room temperature in methylcyclohexane. Then itthe was placed into a cryostat and cooled to 190 K. After the spectrum of the initial complex was acquired, the solution from the cryostat was added to the solution of corresponding base (pyridine, HMPA, DBU; 1–70 eq., c = 0.003–0.21 M dissolved in a small amount of solvent kept at 190 K in liquid nitrogen/*i*PrOH slush bath). The mixture obtained was quickly returned into the cryostat, and the IR spectra were monitored in the 190–290 K temperature range.

Solid *fac*–[(dppm)Mn(CO)_3_H] (**3**, m = 10.0 mg, n = 0.02 mmol) and KHMDS (m = 11.5 mg, n = 0.10 mmol) were placed in separate Schlenk tubes under inert atmosphere. The complex was dissolved in THF or acetonitrile at room temperature, and a small aliquot was taken to IR cell. After the spectrum of the initial complex was acquired, the Schlenk tubes with solution and solid KHMDS were cooled to 243 K in a liquid nitrogen/ethanol slush bath. Then the solution of complex **3** was quickly transferred to KHMDS with a Pasteur pipette, and the obtained mixture was stirred and transferred into the IR cell at room temperature for spectrum acquisition.

For Variable Temperature NMR Studies

Solid *fac*–[(dppm)Mn(CO)_3_H] (**3**, m = 20 mg, n = 0.04 mmol) and KHMDS (m = 23 mg, n = 0.20 mmol) were placed in separate Schlenk tubes under inert atmosphere. The complex was dissolved in THF-*d*_8_ or CD_3_CN at room temperature. After that, the NMR tube under inert atmosphere and Schlenk tubes with solution and solid KHMDS were cooled to 243 K in a liquid nitrogen/ethanol slush bath. The solution of complex **3** was transferred to KHMDS with a Pasteur pipette, and the obtained mixture was stirred and quickly filtered through glass cotton directly into precooled NMR tube. The resulting NMR sample was inserted into a pre-cooled NMR probe at 243 K and then monitored with multi-nuclear NMR spectroscopy at 243 K.

### 3.2. General Procedure for the Interaction with Lewis Acid

For Variable Temperature NMR Studies

The chosen amount of *mer*,*trans*–[(P(OPh)_3_)_2_Mn(CO)_3_H] (**1**, m = 5.3 mg, n = 0.007 mmol) was dissolved in toluene-*d*_8_ at room temperature and monitored in the 200–293 K temperature range. Then the solution of B(C_6_F_5_)_3_ (7 eq., m = 25 mg, n = 0.007 mmol in 0.5 mL toluene-*d_8_*) was added at 200 K, and the reaction mixture was monitored at 200–293 K.

Similarly, complexes **2**–**4**, (m = 20 mg, n = 0.04 mmol) were dissolved in CD_2_Cl_2_, placed into an NMR tube and frozen in liquid nitrogen. Then the solution of the Lewis acid (B(C_6_F_5_)_3_; 1 eq., n = 0.04 mmol) in CD_2_Cl_2_ was poured over the frozen solution in the NMR tube. Two frozen solutions were simultaneously melted in a slush nitrogen/EtOH bath at 180 K, and then the mixture obtained was monitored in the 183–293 K temperature range.

For Variable Temperature IR Studies

The solution of *mer*,*trans*–[(P(OPh)_3_)_2_Mn(CO)_3_H] (**1**, c = 0.005 M) was prepared at room temperature in toluene. Then it was placed into a cryostat and cooled to 190 K. After the spectrum of the initial complex was acquired, the solution from the cryostat was added to B(C_6_F_5_)_3_ (10 eq., c = 0.05 M) and dissolved in a small amount of solvent kept at 190 K in a nitrogen/*i*PrOH slush bath. The obtained mixture was quickly returned to the cryostat and monitored in the 190–290 K temperature range.

The solutions of complexes **2**–**4** (c = 0.003 M) were prepared at room temperature in *n*BuCl (CH_2_Cl_2_ or toluene). Then, they were placed into a cryostat and cooled to 160 K (180 K or 190 K). After the spectrum of the initial complex was acquired, the solution from the cryostat was added to the corresponding Lewis acid B(C_6_F_5_)_3_ (1–1.3 eq., c = 0.003–0.004 M), dissolved in a small amount of solvent and cooled to 160 K (180 K or 190 K) in a nitrogen/EtOH (nitrogen/^i^PrOH) slush bath. The obtained mixture was quickly returned to the cryostat and monitored in the 160 (180 or 190)–290 K temperature range.

## 4. Conclusions

In conclusion, we have shown that tricarbonyl manganese hydride complexes exhibit *dual* reactivity under *Lewis* acid or base treatment. Even the complexes with pronounced hydride character (**2**, **3**) can be deprotonated or, *vice versa*, the complex with acidic properties (**1**) can serve as a hydride donor. The Mn–H bond repolarization occurs at the stage of formation of non-covalent intermediates with *Lewis* acid or base as exemplified for complex **1**. The strength of these non-covalent bonds determines whether subsequent hydrogen ion (hydride or proton) transfer will occur. The studies on the complexes bearing bidentate ligands, *fac*–[(L–L′)Mn(CO)_3_H] (**3**, **4**), revealed that their reactions do not follow simple mechanisms but rather involve the *fac*-to-*mer* isomerizations. The hydride abstraction from these hydrides proceeds from the *mer*-derivatives in which the hydride ligand located *trans* to electron-donating phosphine ligand is more reactive relative to *fac*-isomers where hydride is *trans* to CO ligand. Interestingly, the initial deprotonation site of complex **3** in acetonitrile was observed at the CH_2_ bridge of the dppm ligand, providing the expected metal-deprotonated product via proton migration from anionic hydride intermediate. Since anionic or cationic Mn(I) complexes formed as the result of proton or hydride abstraction are potential intermediates of (de)hydrogenation reactions, quantitative values of kinetic hydricity of the hydride complexes obtained herein may correlate with their potential catalytic activity and be useful for further elucidation of reaction mechanisms.

## Figures and Tables

**Table 1:** IR and ^31^P{^1^H} NMR data for neutral, cationic and anionic manganese species **1**–**4** in different solvents.

Complex	ν_CO_ (cm^−1^)	δ_P_ (ppm)
**1**, [(P(OPh)_3_)_2_Mn(CO)_3_H]	2040 w, 2028 w, 1956 s ^a^ 2041 w, 2028 w, 1955 s ^b^ 2043 w, 2031 w, 1958 s ^c^	183.3
**1^+^**, [(P(OPh)_3_)_2_Mn(CO)_3_][HB(C_6_F_5_)_3_]	2011 s, 1969 s ^a^	153.5
**1^−^**, [(P(OPh)_3_)_2_Mn(CO)_3_][HDBU]	1815 s ^c^	206.5
**2**, [(PPh_3_)_2_Mn(CO)_3_H]	1908 s, 1900 s ^f^	80.5
**2^+^**, [(PPh_3_)_2_Mn(CO)_3_]^+^[HB(C_6_F_5_)_3_]^−^	1980 s, 1930 s ^f^	61.8
**2^−^**, [(PPh_3_)_2_Mn(CO)_3_]^−^[K^+^]	1770 s, 1741 s ^e^	-
**3**, [(dppm)Mn(CO)_3_H]	1996 s, 1916 s, 1909 s ^a^ 1993 s, 1914 s, 1903 s ^b^	30.1 ^a^ 31.8 ^b^
*fac*-**3^+^**, [(dppm)Mn(CO)_3_)][HB(C_6_F_5_)_3_] [27]	2040 s, 1973 s, 1935 s ^d^	10.1
*mer*-**3^+^**, [(dppm)Mn(CO)_3_)][HB(C_6_F_5_)_3_] [27]	2060 s, 2003 s ^d^	10.6, 7.1
**3^CH−^**, *fac*–[(CH**^−^**-dppm)Mn(CO)_3_H][K^+^]	1957 s, 1871 s, 1876 s ^e^ 1956 s, 1870 s ^b^	10.9 ^a^
**3^Mn−^**, [(dppm)Mn**^−^**(CO)_3_][K^+^]	1867 s, 1779 s ^b^	29.9 ^b^
**4**, *fac*–[(P–NHC)Mn(CO)_3_H]	1989 s, 1909 s, 1889 s ^a^ 1988 s, 1907 s,1888 s ^d^	95.8
*fac*-**4^+^**, [(P–NHC)Mn(CO)_3_][HB(C_6_F_5_)_3_]	2032 s, 1949 s, 1921 s ^d^	78.1, 71.1 ^f^
*mer*-**4^+^**, [(P–NHC)Mn(CO)_3_][HB(C_6_F_5_)_3_]	2038 s, 1968 s, 1942 s ^d^	-

^a^ toluene, ^b^ MeCN, ^c^ MCH, ^d^ *n*BuCl, ^e^ THF, ^f^ CH_2_Cl_2_.

**Table 2:** Relative energies (in kcal/mol) of the complex [**3**^Mn−^][K^+^] formation computed at DFT/ωB97XD/def2-TZVP level. Complex [**3**^Mn−^][K^+^] is taken as a zero.

	Toluene	THF	MeCN
E_el_	+2.8	−0.7	−3.1
ΔH	+5.7	+2.0	−0.2
ΔG	+7.6	+2.5	−0.2

**Table 3:** Effective rate constants for the interaction between **1**–**4** and B(C_6_F_5_)_3_ in *n*BuCl at 220 K and activation parameters of hydride transfer.

MnH	*k_eff_*_220K_, M^−1^·s^−1^	∆H^≠^, kcal/mol	∆S^≠^, cal/(mol·K)	ΔG^≠^_220K_, kcal/mol	ΔG^≠^_298K_, kcal/mol
**1**	0.00008	8.6 ± 0.2	−37 ± 1	16.9 ± 0.1	19.8 ± 0.1
**2**	0.147	4.1 ± 0.5	−43 ± 3	13.6 ± 0.3	17.0 ± 0.3
**3**	0.006	9.4 ± 0.6	−26 ± 3	15.0 ± 0.2	17.0 ± 0.2
**4**	0.706	3.8 ± 0.2	−43 ± 1	13.2 ± 0.1	16.5 ± 0.1

## Data Availability

The data presented in this study are available in the article or Supplementary Materials.

## Supplementary Materials

The following supporting information can be downloaded at: https://www.mdpi.com/article/10.3390/molecules28083368/s1: IR and NMR spectroscopic characterization of Mn(I) hydride, cationic and anionic complexes; crystal data and structure refinement parameters; details of thermodynamic and kinetic parameters determination.

## Sample Availability

Samples of the compounds are not available from the authors.
