# Supplementary material for: The Dichotomy of Mn–H Bond Cleavage and Kinetic Hydricity of Tricarbonyl Manganese Hydride Complexes
Source: Molecules. 2023 Apr 11;28(8):3368. doi: 10.3390/molecules28083368 (PMC10143952; doi:10.3390/molecules28083368)
Supplement: Supplementary file 1 [file molecules-28-03368-s001.zip › molecules-2317985-supplementary.pdf]

The Dichotomy of Mn–H Bond Cleavage and Kinetic Hydricity of Tricarbonyl Manganese Hydride Complexes

Elena Osipova[1], Sergey Kovalenko[1], Ekaterina Gulyaeva[1,2], Nikolay Kireev[1], Alexander Pavlov[1], Oleg Filippov[1], Anastasia Danshina[1,3], Dmitry Valyaev[2]*, Yves Canac[2], Elena Shubina[1]*, Natalia Belkova[1]*

1 A.N. Nesmeyanov Institute of Organoelement Compounds, Russian Academy of Sciences (INEOS RAS), 28, Vavilova Str., 119334 Moscow, Russia; aosipova92@gmail.com (E.S.O.); kovalenko2000as@gmail.com (S.A.K.); elenor.kagami@gmail.com (E.S.G.); koly-100@mail.ru (N.V.K.); alex90pavlov@mail.ru (A.A.P.);

h-bond@ineos.ac.ru (O.A.F.); danshina.aa@phystech.edu (A.A.D.); shu@ineos.ac.ru (E.S.S.)

2 LCC-CNRS, Université de Toulouse, CNRS, 205 Route de Narbonne, 31077 Toulouse, CEDEX 4, France; yves.canac@lcc-toulouse.fr

3 Center of National Technological Initiative, Bauman Moscow State Technical University, 2nd Baumanskaya Str., 5, 105005 Moscow, Russia

4 Moscow Institute of Physics and Technology, Institutskiy per., 9,

141700 Dolgoprudny, Russia

* Correspondence: dmitry.valyaev@lcc-toulouse.fr (D.A.V.); nataliabelk@ineos.ac.ru (N.V.B.)

Electronic Supplementary Information

Table of contents

Figure S1. IR spectra of complex 1 (c=0.003M) and after HMPA (1-10 equiv.) (*left*) and pyridine (1, 10, 50, 100 equiv.) (*right*) adding. 190K, l = 0.1 cm, methylcyclohexane. 2

Figure S2. IR spectra of complex 3 (c=0.003M) and after HMPA (40 and 70 equiv.) adding. 190K, l = 0,05 cm, toluene. 2

Figure S3. Time evolution of IR spectra for ν_{co} of complex 1 after addition of DBU (1.1 equiv.) during 1.2 h. *c*(1) = 0.003 M, 230 K, l = 0.1 cm, methylcyclohexane. IR spectrum of 1 alone (light blue) is shown for comparison. 2

Thermodynamic data for proton transfer between 1 and DBU from IR study. 3

Determination of kinetic parameters of proton transfer between 1 and DBU from IR study. 4

Figure S4. IR spectra of complex 1 (c = 0.003 M) and after $[Bu_4N]^+[4\text{-}NO_2C_6H_4O]^-$ addition (1 equiv.). 298 K, l = 0.1 cm, MeCN. 4

Figure S5. IR spectra of complex 2 (*c* = 0.0144 M; blue line) and 2 after KHMDS addition (2 equiv.; red line). 298 K, l = 0.1 cm, THF. 5

Figure S6. $^{31}P\{^1H\}$ NMR spectra (162.0 MHz) of complex 3 and its reaction with KHMDS (1:5) in THF-d_8 at 243 K (*left*) and in CD_3CN at 263 K (*right*). 5

Figure S7. IR spectra of complex 4 (c = 0.0033 M) and its mixture with $B(C_6F_5)_3$ (c = 0.0037 M). BuCl, 160-290K, l = 0.05 cm. 5

Figure S8. Proposed reaction pathway between hydride complex 4 and BAr_3. Coordinated solvent molecules to cationic species are omitted. 6

Table S1. $^{31}P\{^1H\}$ NMR chemical shifts for complexes 4a and 4b in different media. 6

Figure S9. Top: IR spectra of 1 (c = 0.005 M) and its mixture with $B(C_6F_5)_3$ (10 equiv). Toluene, l = 0,05 cm, 190-290K (step 10K). Bottom: temperature dependence of intensity for ν_{CO} bands of 1 and 1···LA and 1^+ 7

Figure S10. ^1H (*left*) and $^{31}P\{^1H\}$ (*right*) NMR spectra of 1 (*c* = 0.01M; bottom) and its mixture with $B(C_6F_5)_3$ (10 equiv.). Toluene-d_8, 200-290 K. 7

Figure S11. IR spectra of complex 2 (c = 0.003 M) and its mixture with $B(C_6F_5)_3$ (*c* = 0.003 M). CH_2Cl_2, 180-290K, l = 0.05 cm. 8

Kinetic study of the hydrogen abstraction from complexes 1 – 4 to Lewis acid 8

Figure S12. Plot for the determination of effective rate constant (k_{eff}) of 4 with $B(C_6F_5)_3$ in *n*BuCl at 170 K 8

Figure S13. Plot $-\ln(k_{eff}\cdot h/k_b\cdot T)$ vs 1/T for the reaction of 4 with $B(C_6F_5)_3$ in *n*BuCl (160 - 200 K). 8

Table S2. Crystal data and structure refinement parameters for $[(P\text{-}NHC)Mn(CO)_3(MeCN)][BF_4]$ (4^{MeCN}) and $[(PPh_3)_2Mn(CO)_3(MeCN)][BF_4]$ (2^{MeCN}). 9

NMR spectroscopic characterization of Mn(I) cationic and anionic complexes (Figure S14-S26) 10

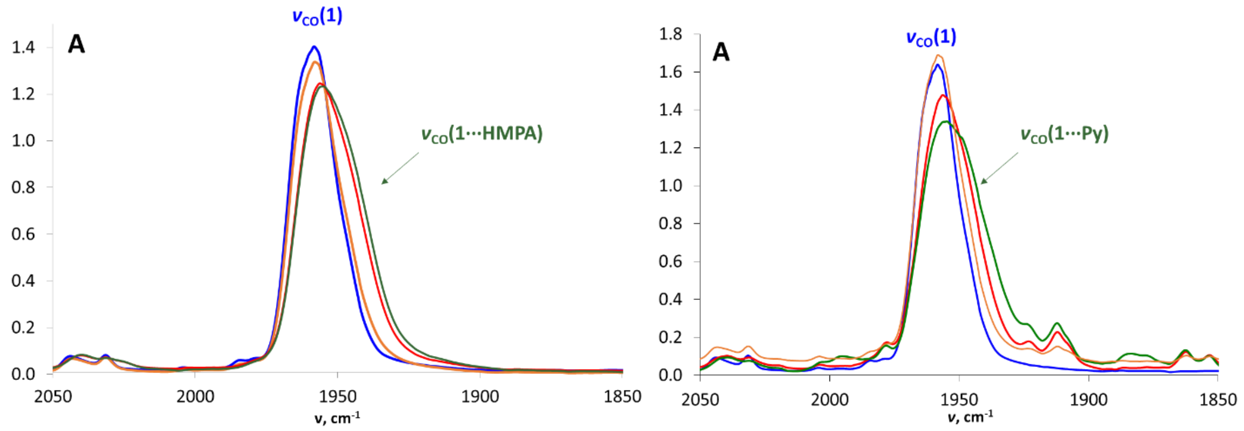

Figure S1. IR spectra of complex **1** (c=0.003M) and after HMPA (1-10 equiv.) (*left*) and pyridine (1, 10, 50, 100 equiv.) (*right*) adding. 190K, l = 0.1 cm, methylcyclohexane.

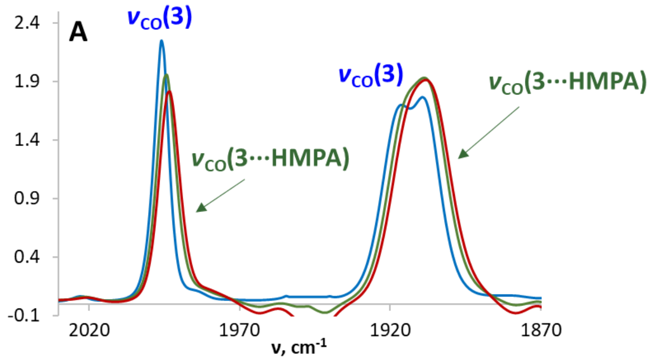

Figure S2. IR spectra of complex **3** (c=0.003M) and after HMPA (40 and 70 equiv.) adding. 190K, l = 0,05 cm, toluene.

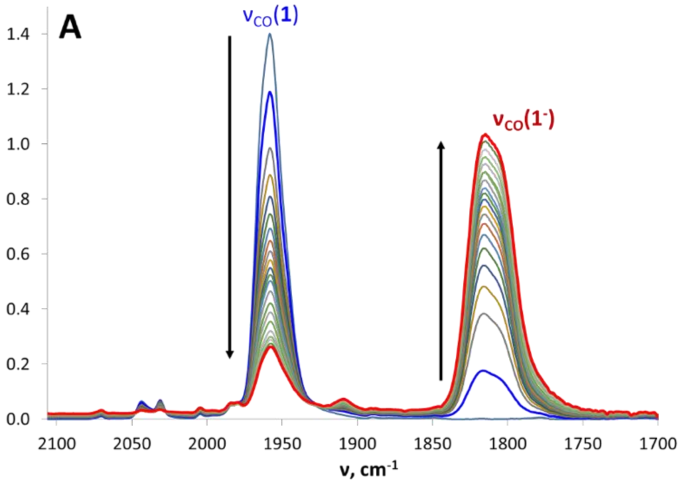

Figure S3. Time evolution of IR spectra for ν_{co} of complex **1** after addition of DBU (1.1 equiv.) during 1.2 h. *c*(**1**) = 0.003 M, 230 K, l = 0.1 cm, methylcyclohexane. IR spectrum of **1** alone (light blue) is shown for comparison.

Thermodynamic data for proton transfer between 1 and DBU from IR study.

For the reaction of $[P(OPh)_3]_2Mn(CO)_3H$ (**1**) and 1,8-diazabicyclo[5.4.0]undec-7-ene (DBU) in MCH experimental equilibrium constants were obtained at 230 K, 240 K, 250 K and thermodynamic parameters (ΔH^o, ΔS^o, ΔG^o_{298K}) of proton transfer were obtained by Van't-Hoff method.

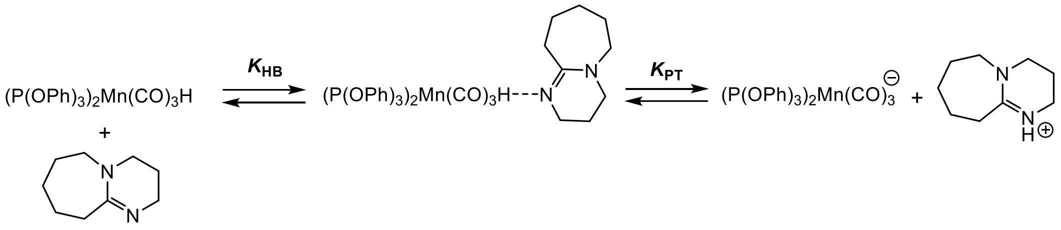

$$K_{exp} = K_{HB} \cdot K_{PT} = \frac{[1]^{-}[\mathrm{DBU}H]^{+}}{[1][\mathrm{DBU}]}$$

$$\Delta G^o = \Delta H^o - T\Delta S^o$$

$$-\frac{\Delta H^o}{T} + \Delta S^o = RlnK_{exp}$$

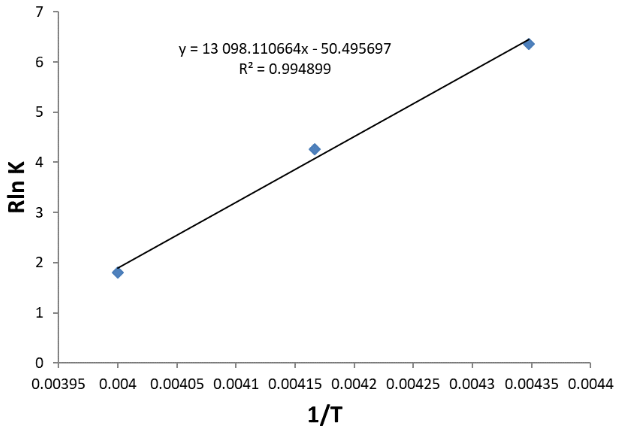

T, K	1/T	K_{exp}	RlnK_{exp}
250	0.004	2.47	1.80
240	0.004167	8.54	4.27
230	0.004348	24.46	6.36

Thermodynamic parameters	
ΔH^o, kcal/mol	13.1±0.9
ΔS^o, cal/(mol·K)	50±4
ΔG^o_{298K}, kcal/mol	1.9±0.2

Determination of kinetic parameters of proton transfer between 1 and DBU from IR study.

The observed rate constants (k_{obs}) of proton transfer from the complex $[P(OPh)_3]_2Mn(CO)_3H$ (**1**) to DBU were obtained at 190 K, 210 K and 230 K by IR monitoring (ν_{CO}(**1**) decrease). By use of Eyring equation, the activation parameters ($\Delta H^{\neq}$, $\Delta S^{\neq}$, $\Delta G^{\neq}_{298K}$) were determined.

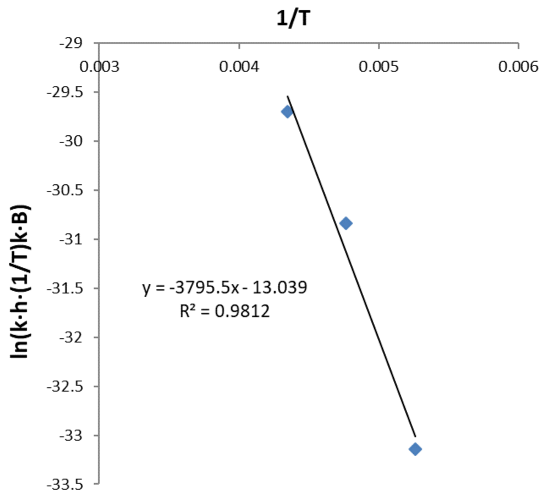

T, K	1/T	k_{obs}	Ln(k*ħ*(1/T)/k_B)
190	0.005263	0.016	-33.14
210	0.004762	0.177	-30.84
230	0.004348	0.609	-29.69

Activation parameters	
$\Delta H^{\neq}$, kcal/mol	7.5±0.5
$\Delta S^{\neq}$, cal/(mol·K)	26±2
$\Delta G^{\neq}_{298K}$, kcal/mol	15.3±0.2

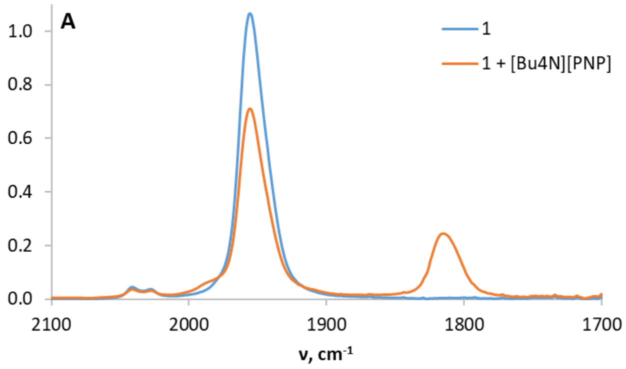

Figure S4. IR spectra of complex **1** (c = 0.003 M) and after $[Bu_4N]^+[4\text{-}NO_2C_6H_4O]^-$ addition (1 equiv.). 298 K, l = 0.1 cm, MeCN.

pK_a value was calculated with following equation:

$-pK_{eq} = pK_a(BH^+) - pK_a(MH)$ [1];

K_{eq} = 0.25 was obtained from the spectral data (Figure S4); pK_a(BH^+) = 20.7 in MeCN [2].

[1] R. T. Edidin, J. M. Sullivan and J. R. Norton, *J. Am. Chem. Soc.*, **1987**, *109*, 3945-3953.

[2] F. Eckert, I. Leito, I. Kaljurand, A. Kütt, A. Klamt and M. Diedenhofen, *J. Comput. Chem.*, **2009**, *30*, 799-810.

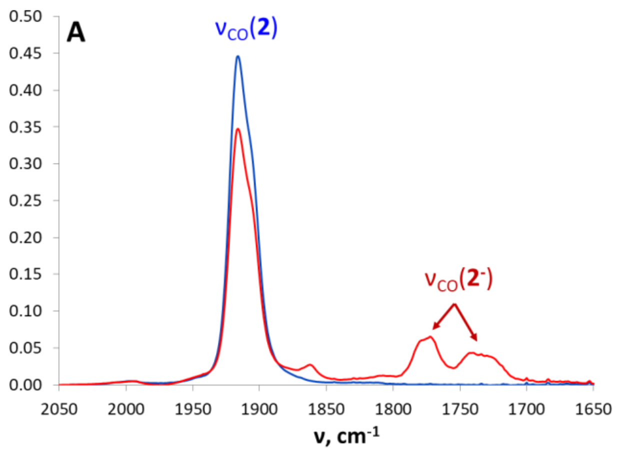

Figure S5. IR spectra of complex **2** (*c* = 0.0144 M; blue line) and **2** after KHMDS addition (2 equiv.; red line). 298 K, l = 0.1 cm, THF.

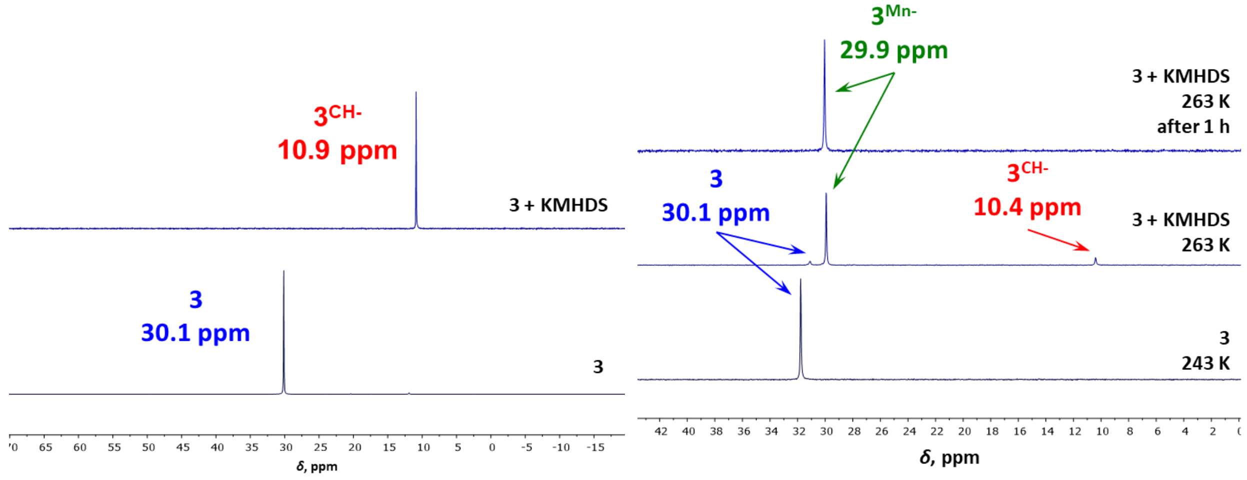

Figure S6. $^{31}P\{^1H\}$ NMR spectra (162.0 MHz) of complex **3** and its reaction with KHMDS (1:5) in THF-d_8 at 243 K (*left*) and in CD_3CN at 263 K (*right*).

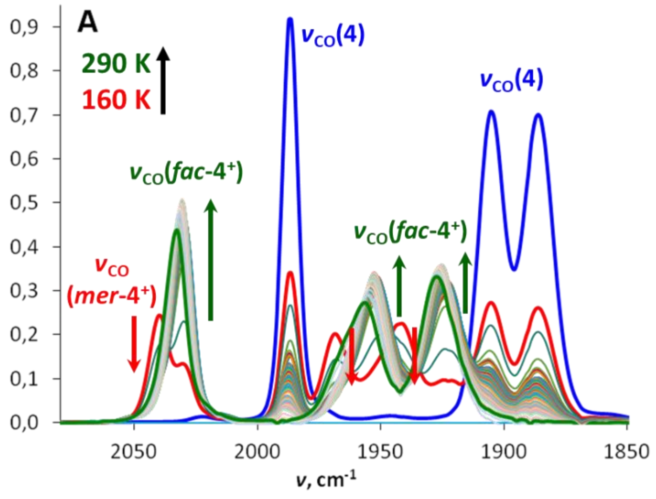

Figure S7. IR spectra of complex **4** (c = 0.0033 M) and its mixture with $B(C_6F_5)_3$ (c = 0.0037 M). BuCl, 160-290K, l = 0.05 cm.

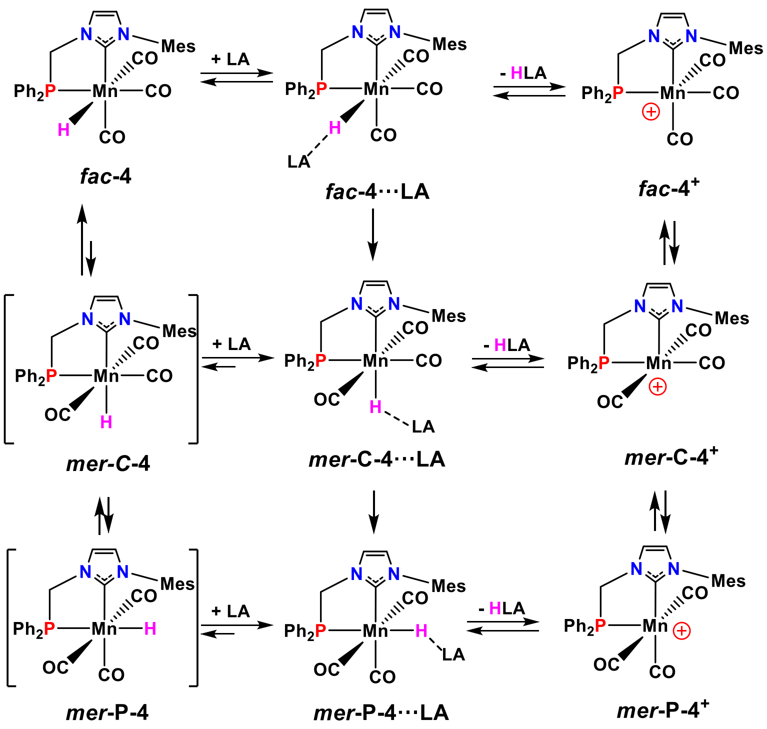

Figure S8. Proposed reaction pathway between hydride complex **4** and BAr_3. Coordinated solvent molecules to cationic species are omitted.

Table S1. $^{31}P\{^{1}H\}$ NMR chemical shifts for complexes **4a** and **4b** in different media.

Solvent	δ_P (**4a**)	δ_P (**4b**)
Toluene	74.1	71.1
BuCl	76.1	71.5
C_6H_5Cl	77.7	71.1
CD_2Cl_2	78.1	71.1
MeCN	77.5	-

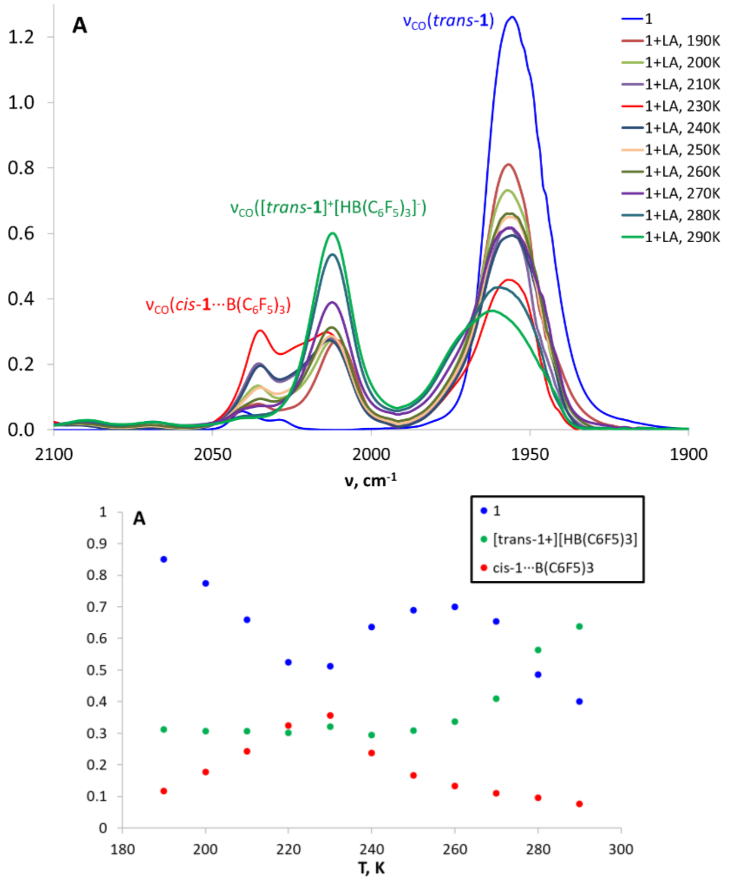

Figure S9. Top: IR spectra of **1** (c = 0.005 M) and its mixture with $B(C_6F_5)_3$ (10 equiv). Toluene, l = 0,05 cm, 190-290K (step 10K). Bottom: temperature dependence of intensity for ν_{CO} bands of **1** and **1**···LA and **1**$^+$.

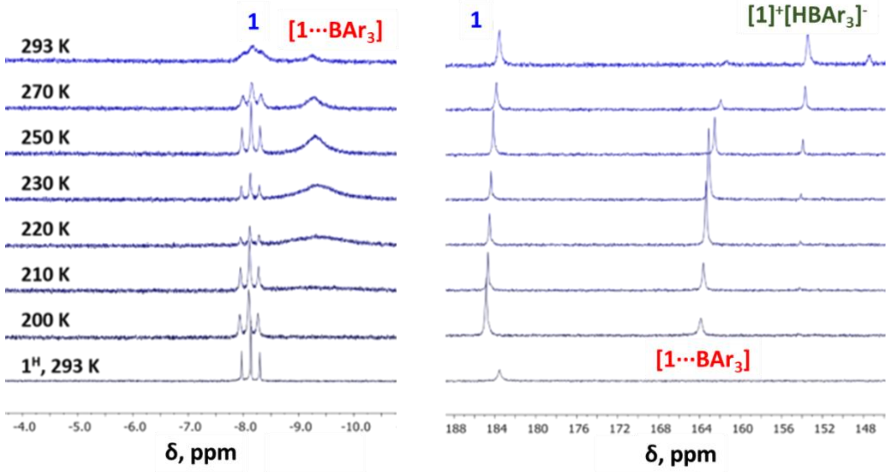

Figure S10. ^{1}H (*left*) and ^{31}P{^{1}H} (*right*) NMR spectra of **1** (*c* = 0.01M; bottom) and its mixture with $B(C_6F_5)_3$ (10 equiv.). Toluene-d_8, 200-290 K.

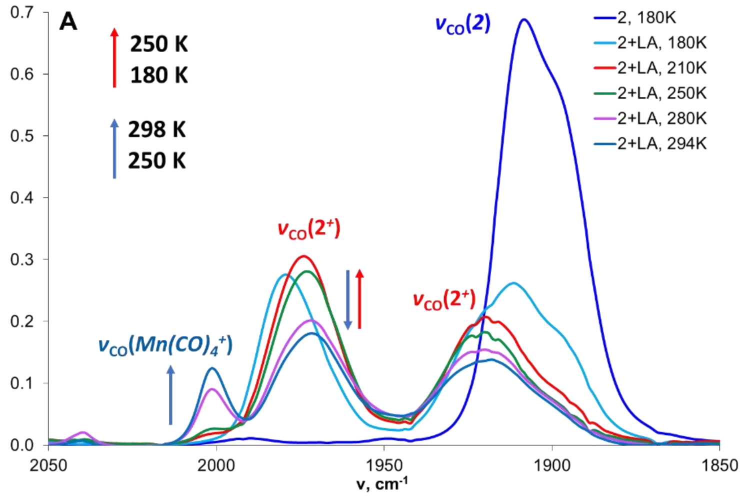

Figure S11. IR spectra of complex **2** (c = 0.003 M) and its mixture with $B(C_6F_5)_3$ (*c* = 0.003 M). CH_2Cl_2, 180-290K, l = 0.05 cm.

Kinetic study of the hydrogen abstraction from complexes 1 – 4 to Lewis acid

For the hydride abstraction reaction from the complexes **1** – **4** to $B(C_6F_5)_3$ current concentrations of the components were calculated from the absorptions obtained by IR monitoring (ν_{CO} of the initial hydride decrease) at the temperature range (160 – 200 K). The effective rate constants (k_{eff}) were obtained by second-order law for reaction type A + B → C + B:

$$k = \frac{1}{t(a_o - b_0)} ln \frac{b_0(a_0 - a)}{a_0(b_0 - b)}$$

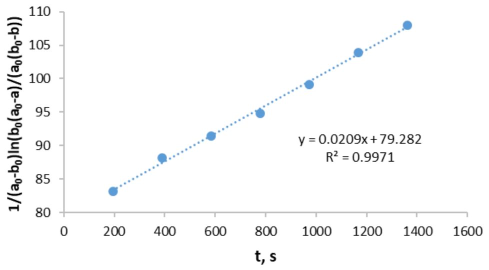

Figure S12. Plot for the determination of effective rate constant (k_{eff}) of **4** with $B(C_6F_5)_3$ in *n*BuCl at 170 K.

By use of Eyring equation, the activation parameters ($\Delta H^{\neq}$, $\Delta S^{\neq}$, $\Delta G^{\neq}_{298K}$) were determined at 160 – 200 K temperature range:

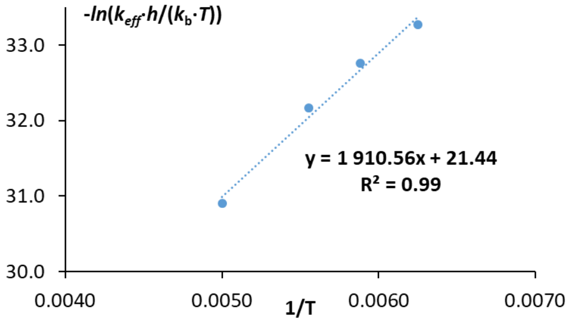

Figure S13. Plot $-\ln(k_{eff}\cdot h/k_b\cdot T)$ vs 1/T for the reaction of **4** with $B(C_6F_5)_3$ in *n*BuCl (160 - 200 K).

Table S2. Crystal data and structure refinement parameters for [(P-NHC)Mn$(CO)_3$(MeCN)][BF_4] (**4**^{MeCN}) and [$(PPh_3)_2$Mn$(CO)_3$(MeCN)][BF_4] (**2**^{MeCN}).

Complex	[(P-NHC)Mn$(CO)_3$(MeCN)][BF_4] **4**^{MeCN}	[$(PPh_3)_2$Mn$(CO)_3$(MeCN)][BF_4] **2**^{MeCN}
Empirical formula	$C_{30}H_{28}BF_4MnN_3O_3P$	$C_{42}H_{35}BCl_2F_4MnNO_3P_2$
Formula weight	651.27	876.30
T, K	100	100
Crystal system	Monoclinic	Monoclinic
Space group	P-1	$P2_1/c$
Z	4	4
a, Å	12.8975(3)	11.802(3)
b, Å	15.9475(3)	25.753(8)
c, Å	16.7390(4)	13.846(3)
α, °	73.6000(10)	90
β, °	89.9020(10)	107.127(8)
γ, °	66.2240(10)	90
V, Å^3	2997.78(12)	4021.9(19)
D_{calc} (g cm^{-1})	1.443	1.447
μ, cm^{-1}	5.55	6
F(000)	1336	1792
$2\theta_{max}$, °	58	60
Reflections measured	40707	43067
Independent reflections	15881	11717
Observed reflections [$I > 2\sigma(I)$]	9234	8607
Parameters	805	506
R1	0.0693	0.0528
wR2	0.1876	0.1238
GOOF	1.031	1.089
$\Delta\rho_{max}$/ $\Delta\rho_{min}$ (e Å^{-3})	1.451/-0.580	0.597/-0.781

NMR spectroscopic characterization of Mn(I) cationic and anionic complexes

1, *mer,trans*-[$(P(OPh)_3)_2Mn(CO)_3H$] in CD_3CN:

^1H NMR (400.1 MHz, 293 K, CD_3CN): δ 7.34 (t, $^3J_{HH}$ = 7.8 Hz, 12H, CH_{Ar}), 7.20 (t, $^3J_{HH}$ = 7.3 Hz, 6H, CH_{Ar}), 7.12 (d, $^3J_{HH}$ = 8.0 Hz, 12H, CH_{Ar}), −8.60 (t, $^2J_{PH}$ = 49.2 Hz, 1H, Mn-*H*).

$^{31}P\{^1H\}$ NMR (162.0 MHz, 293 K, CD_3CN): δ 183.3 (s)

1, *mer,trans*-[$(P(OPh)_3)_2Mn(CO)_3H$] in toluene-d_8:

^1H NMR (400.1 MHz, 293 K, toluene-d_8): δ 7.17 (d, $^3J_{HH}$ = 8.0 Hz, 12H, CH_{Ar}), 6.97 (t, $^3J_{HH}$ = 7.7 Hz, 12H, CH_{Ar}), 6.83 (t, $^3J_{HH}$ = 7.4 Hz, 6H, CH_{Ar}), −8.23 (t, $^2J_{PH}$ = 49.8 Hz, 1H, Mn-*H*).

$^{31}P\{^1H\}$ NMR (162.0 MHz, 293 K, toluene-d_8): δ 183.4 (s)

1$^-$, [$(P(OPh)_3)_2Mn(CO)_3$][HDBU] in CD_3CN:

^1H NMR (400.1 MHz, 293 K, CD_3CN): δ 7.73-7.39 (m, 30H, CH_{Ar})

$^{31}P\{^1H\}$ NMR (162.0 MHz, 293 K, CD_3CN): δ 206.5 (s)

1$^+$, [$(P(OPh)_3)_2Mn(CO)_3$][$HB(C_6F_5)_3$] in toluene-d_8:

^1H NMR (400.1 MHz, 293 K, toluene-d_8): δ 6.80-6.94 (m, 30H, CH_{Ar})

$^{31}P\{^1H\}$ NMR (162.0 MHz, 293 K, toluene-d_8): δ 153.5 (s)

2, *mer,trans*-[$(PPh_3)_2Mn(CO)_3H$] in CD_2Cl_2:

^1H NMR (400.1 MHz, 293 K, CD_2Cl_2): δ 7.54 (m, 12H, *o*-CH_{Ar}), 7.42 (br t, 18H, *m*- and *p*-CH_{Ar}), −7.38 (t, $^2J_{HP}$ = 27.4 Hz, 1H, Mn-*H*).

$^{31}P\{^1H\}$ NMR (162.0 MHz, 293 K, CD_2Cl_2): δ 80.5 (s).

2$^+$, [$(PPh_3)_2Mn(CO)_3$][$HB(C_6F_5)_3$]:

^1H NMR (400.1 MHz, 293 K, CD_2Cl_2): δ 7.55 – 7.42 (m, 30H, CH_{Ar}).

$^{31}P\{^1H\}$ NMR (162.0 MHz, 293 K, CD_2Cl_2): δ 61.8 (br s).

3, *fac*-[(dppm)$Mn(CO)_3H$] in THF-d_8:

^1H NMR (400.1 MHz, 243 K, THF-d_8): δ 7.70 (br s, 4H, CH_{Ar}), 7.63 (br s, 4H, CH_{Ar}), 7.42 (br s, 12H, CH_{Ar}), 4.42 (dtd, $^2J_{HH}$ = 15.5 Hz, $^2J_{PH}$ = 9.5 Hz, $^4J_{HH}$ = 5.5 Hz, 1H, PCH_2P), 4.07 (dt, $^2J_{HH}$= 15.4 Hz, $^2J_{PH}$ = 11.3 Hz, 1H, PCH_2P), −5.53 (td, $^2J_{PH}$ = 44.0 Hz, $^4J_{HH}$ = 5.5 Hz, 1H, Mn−*H*).

$^{31}P\{^1H\}$ NMR (162.0 MHz, 243 K, THF-d_8): δ 30.1 (s).

$^{13}C\{^1H\}$ NMR (100.6 MHz, 243 K, THF-d_8): δ 225.9 (t, $^2J_{CP}$ = 7.0 Hz, Mn–*C*O), 222.7 (t, $^2J_{CP}$ = 13.2 Hz, Mn–*C*O), 138.8 (vt, J_{PC} = 24.4 Hz, C_{ipso} PPh_2), 136.6 (vt, J_{PC} = 16.0 Hz, C_{ipso} PPh_2), 132.8–132.7 (m, CH_{Ar}), 131.2 (d, J_{PC} = 5.4 Hz, CH_{Ar}), 129.5 (vdt, J_{PC} = 10.7, 5.0 Hz, CH_{Ar}), 48.0 (t, $^1J_{CP}$ = 22.4 Hz, PCH_2P).

3^{CH-}, *fac*-[(CH^--dppm)$Mn(CO)_3H$] in THF-d_8:

^1H NMR (400.1 MHz, 243 K, THF-d_8): δ 7.74 (br s, 4H, CH_{Ar}), 7.67 (br s, 4H, CH_{Ar}), 7.69–7.65 (m, 12H, CH_{Ar}), 1.95 (t, $^2J_{PH}$= 5.0 Hz, 1H, PCH^-P), −5.54 (t, $^2J_{PH}$ = 44.0 Hz, 1H, Mn−*H*).

$^{31}P\{^1H\}$ NMR (162.0 MHz, 243 K, THF-d_8): δ 10.9 (s).

$^{13}C\{^1H\}$ NMR (100.6 MHz, 243 K, THF-d_8): δ 231.8 (t, $^2J_{CP}$ = 7.1 Hz, Mn–*C*O), 225.0 (t, $^2J_{CP}$ = 14.7 Hz, Mn–*C*O), 150.0 (vt, J_{PC} = 20.9 Hz, C_{ipso} PPh_2), 149.0 (vt, J_{PC} = 18.8 Hz, C_{ipso} PPh_2), 132.1 (vt, J_{PC} = 4.9 Hz, CH_{Ar}), 131.7 (vt, J_{PC} = 5.1 Hz, CH_{Ar}), 127.6–127.5 (m, CH_{Ar}), 127.1 (s, CH_{Ar}), 20.8 (t, $^1J_{CP}$ = 51.4 Hz, PCH^-P).

3, *fac*-[(dppm)$Mn(CO)_3H$] in CD_3CN:

^1H NMR (400.1 MHz, 243 K, CD_3CN): δ 7.66–7.58 (m, 8H, CH_{Ar}), δ 7.44–7.40 (br s, 12H, CH_{Ar}), 4.61 (dtd, $^2J_{HH}$ = 15.6 Hz, $^2J_{PH}$ = 11.2 Hz, $^4J_{HH}$ = 5.6 Hz, 1H, PCH_2P), 3.99 (dt, $^2J_{HH}$= 15.6 Hz, $^2J_{PH}$ = 11.5 Hz, 1H, PCH_2P), −5.09 (td, $^2J_{PH}$ = 43.4 Hz, $^4J_{HH}$ = 5.7 Hz, 1H, Mn−*H*).

$^{31}P\{^1H\}$ NMR (162.0 MHz, 243 K, CD_3CN): δ 31.8 (s).

$^{13}C\{^1H\}$ NMR (100.6 MHz, 243 K, CD_3CN): δ 225.5 (t, $^2J_{CP}$ = 7.0 Hz, Mn–*C*O), 222.8 (t, $^2J_{CP}$ = 15.2 Hz, Mn–*C*O), 137.9 (vt, J_{PC} = 24.6 Hz, C_{ipso} PPh_2), 135.0 (vt, J_{PC} = 16.7 Hz, C_{ipso} PPh_2), 132.3 (vt, J_{PC} = 5.8 Hz, CH_{Ar}), 132.0 (vt, J_{PC} = 5.6 Hz, CH_{Ar}), 131.2 (d, J_{PC} = 6.3 Hz, CH_{Ar}), 129.6 (vdt, J_{PC} = 10.5, 4.9 Hz, CH_{Ar}), 48.0 (t, $^1J_{CP}$ = 23.0 Hz, PCH_2P).

$3^{Mn–}$, *fac*-[(dppm)Mn(CO)$_3$](K) in CD_3CN:

^{1}H NMR (400.1 MHz, 293 K, THF-d_8): δ 7.64–7.59 (m, 8H, CH_{Ar}), 7.30–7.21 (m, 12H, CH_{Ar}).

^{31}P{^{1}H} NMR (162.0 MHz, 243 K, THF-d_8): δ 29.9 (s).

^{13}C{^{1}H} NMR (100.6 MHz, 243 K, THF-d_8): δ 245.7 (t, $^2J_{CP}$ = 10.4 Hz, Mn–*C*O), 173.2 (s, C_{ipso} PPh$_2$), 144.6 (vt, J_{PC} = 12.9 Hz, C_{ipso} PPh$_2$), 131.7 (vt, J_{PC} = 5.9 Hz, *C*H$_{Ar}$), 128.7 (s, *C*H$_{Ar}$), 128.4 (vt, J_{PC} = 4.3 Hz, *C*H$_{Ar}$), 45.3 (t, $^1J_{CP}$ = 22.1 Hz, P*C*H$_2$P).

4, *fac*-[(P-NHC)Mn(CO)$_3$H]:

^{1}H NMR (300.1 MHz, 293 K, CD_2Cl_2): δ 7.75 (m, 2H, CH_{Ph}), 7.56 (m, 2H, CH_{Ph}), 7.26 (s, 1H, CH_{Im}), 7.45 (m, 6H, CH_{Ph}), 7.01 (s, 1H, CH_{Im}), 6.99 (s, 1H, CH_{Mes}), 6.87 (s, 1H, CH_{Mes}), 4.86 (dd, $^2J_{HH}$ = 13.2 Hz, $^2J_{PH}$ = 7.3 Hz, 1H, PCH_2), 4.45 (dd, $^2J_{HH}$ = 13.2 Hz, $^2J_{PH}$ = 3.6 Hz, 1H, PCH_2), 2.35 (s, 3H, CH_{3Mes}), 2.04 (s, 3H, CH_{3Mes}), 1.99 (s, 3H, CH_{3Mes}), –7.33 (d, $^2J_{PH}$ = 53.4 Hz, Mn–*H*);

^{31}P{^{1}H} NMR (162.0 MHz, 293 K, CD_2Cl_2): δ 95.8 (s).

4^{MeCN}, *fac*-[(P-NHC)Mn(CO)$_3$(MeCN)]$^+$[BF$_4$]$^-$:

^{1}H NMR (300.1 MHz, 293 K, CD_2Cl_2): 7.93 (s, 1H, CH_{Ph}), 7.64-7.56 (m, 9H, CH_{Ph} + 1H, CH_{Im}), 7.20 (s, 1H, CH_{Im}), 7.06 (s, 1H, CH_{Mes}), 7.05 (s, 1H, CH_{Mes}), 5.50 (dd, $^2J_{HH}$ = 14.5, $^2J_{PH}$ = 6.8 Hz, 1H, PCH_2), 5.00 (dd, $^2J_{HH}$ = 15.0, $^2J_{PH}$ = 5.3 Hz, 1H, PCH_2), 2.36 (s, 3H, CH_{3Mes}), 1.99 (s, 3H, CH_3CN–Mn), 1.93 (s, 3H, CH_{3Mes}), 1.69 (s, 3H, CH_{3Mes}).

^{31}P{^{1}H} NMR (162.0 MHz, 293 K, CD_2Cl_2): δ 77.5 (s).

4a, *fac*-[(P-NHC)Mn(CO)$_3$(CD$_2$Cl$_2$)]$^+$[HB(C$_6$F$_5$)$_3$]$^-$:

^{1}H NMR (300.1 MHz, 293 K, CD_2Cl_2): δ 7.66 (s, 1H, CH_{Ph}), 7.51 (m, 2H, CH_{Ph} + CH_{Im}), 7.39-7.43 (m, 3H, CH_{Ph}), 7.23 (2H, CH_{Ph}), 7.17 (2H, CH_{Ph}), 7.14 (s, 1H, CH_{Im}), (7.05 (s, 1H, CH_{Mes}), 7.02 (s, 1H, CH_{Mes}), 5.07 (dd, $^2J_{HH}$ = 14.0, $^2J_{PH}$ = 5.1 Hz, 1H, PCH_2), 4.94 (dd, $^2J_{HH}$ = 14.1, $^2J_{PH}$ = 6.6 Hz, 1H, PCH_2), 2.37 (s, 3H, CH_{3Mes}), 1.97 (s, 3H, CH_{3Mes}), 1.79 (s, 3H, CH_{3Mes}).

^{31}P{^{1}H} NMR (162.0 MHz, 293 K, CD_2Cl_2): δ 78.1 (s).

4b, *fac*-[(P-NHC)Mn(CO)$_3$]$^+$[HB(C$_6$F$_5$)$_3$]$^-$:

^{1}H NMR (300.1 MHz, 293 K, CD_2Cl_2): δ 7.87-7.81 (m, 2H, CH_{Ph}), 7.51 (m, 3H, CH_{Ph} + CH_{Im}), 7.36 (3H, CH_{Ph}), 7.25 (2H, CH_{Ph}), 7.15 (1H, CH_{Ph}), 7.02 (s, 1H, CH_{Im}), 6.98 (s, 1H, CH_{Mes}), 6.94 (s, 1H, CH_{Mes}), 5.48 (vt, $^2J_{HH}$ = 14.4 Hz, $^2J_{PH}$ = 13.7 Hz, 1H, PCH_2), 4.99 (d, $^2J_{HH}$ = 14.4 Hz, 1H, PCH_2), 2.30 (s, 3H, CH_{3Mes}), 1.92 (s, 3H, CH_{3Mes}), 1.76 (s, 3H, CH_{3Mes}).

^{31}P{^{1}H} NMR (162.0 MHz, 293 K, CD_2Cl_2): δ 71.1 (s).

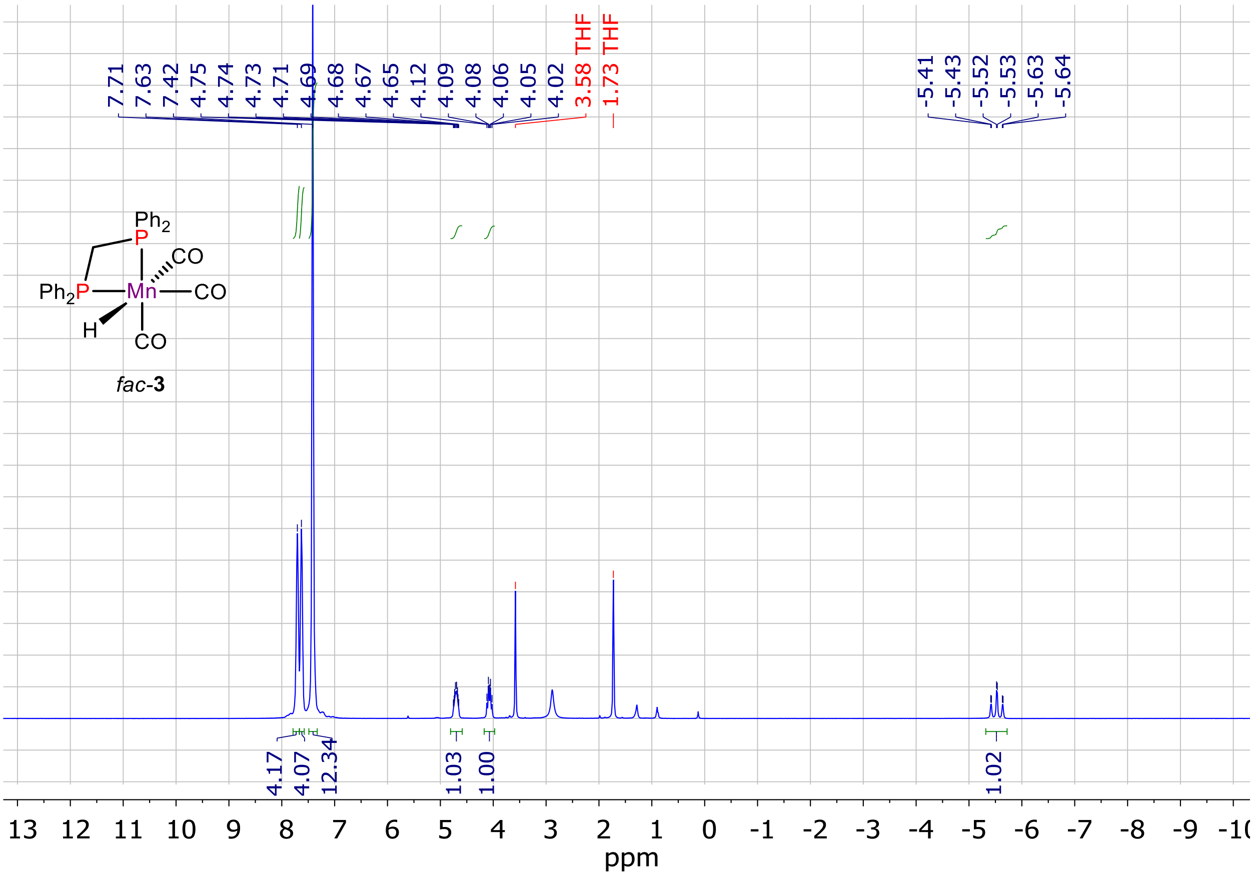

Figure S14. ^{1}H NMR spectrum of complex *fac*-**3** (400.1 MHz, THF-d_8, 298 K).

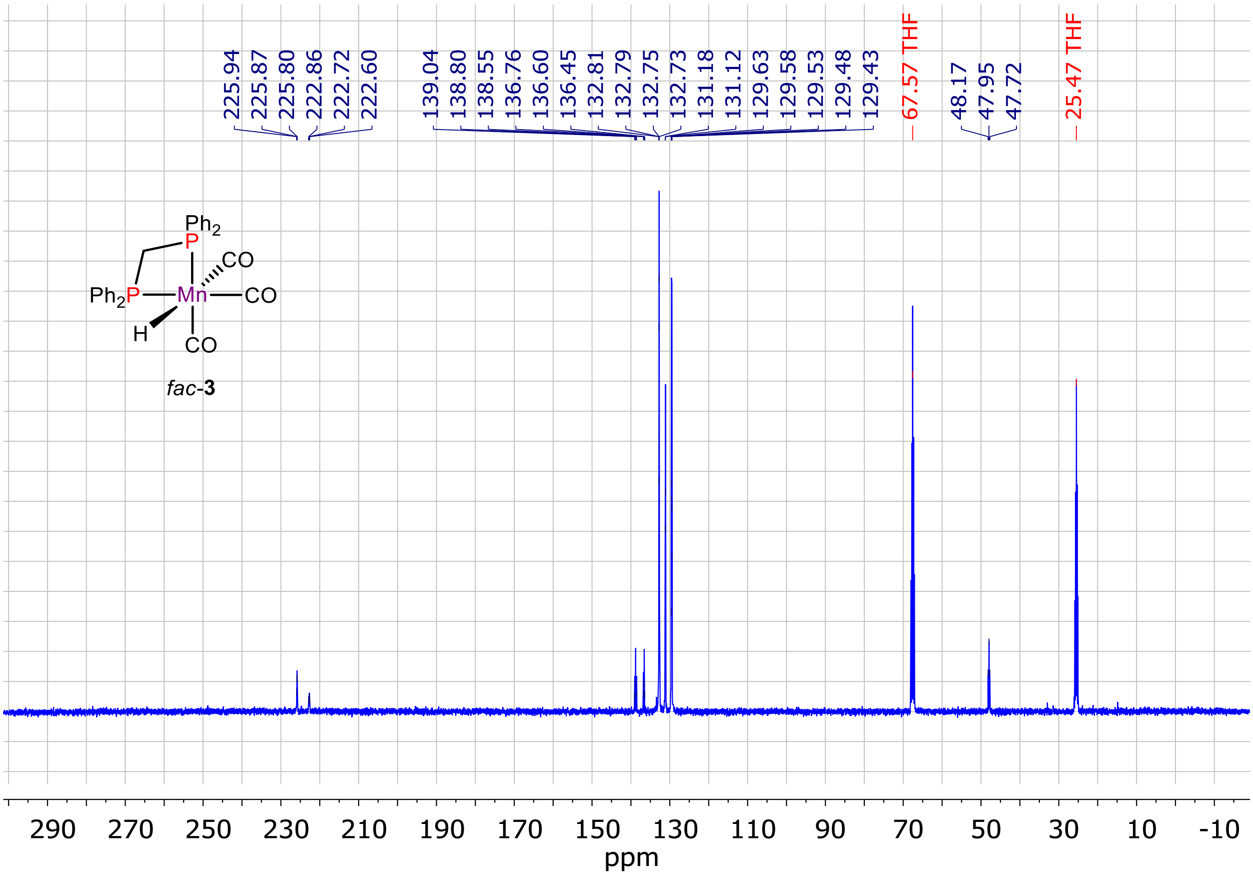

Figure S15. ^{13}C NMR spectrum of complex *fac*-**3** (150.9 MHz, THF-d_8, 298 K).

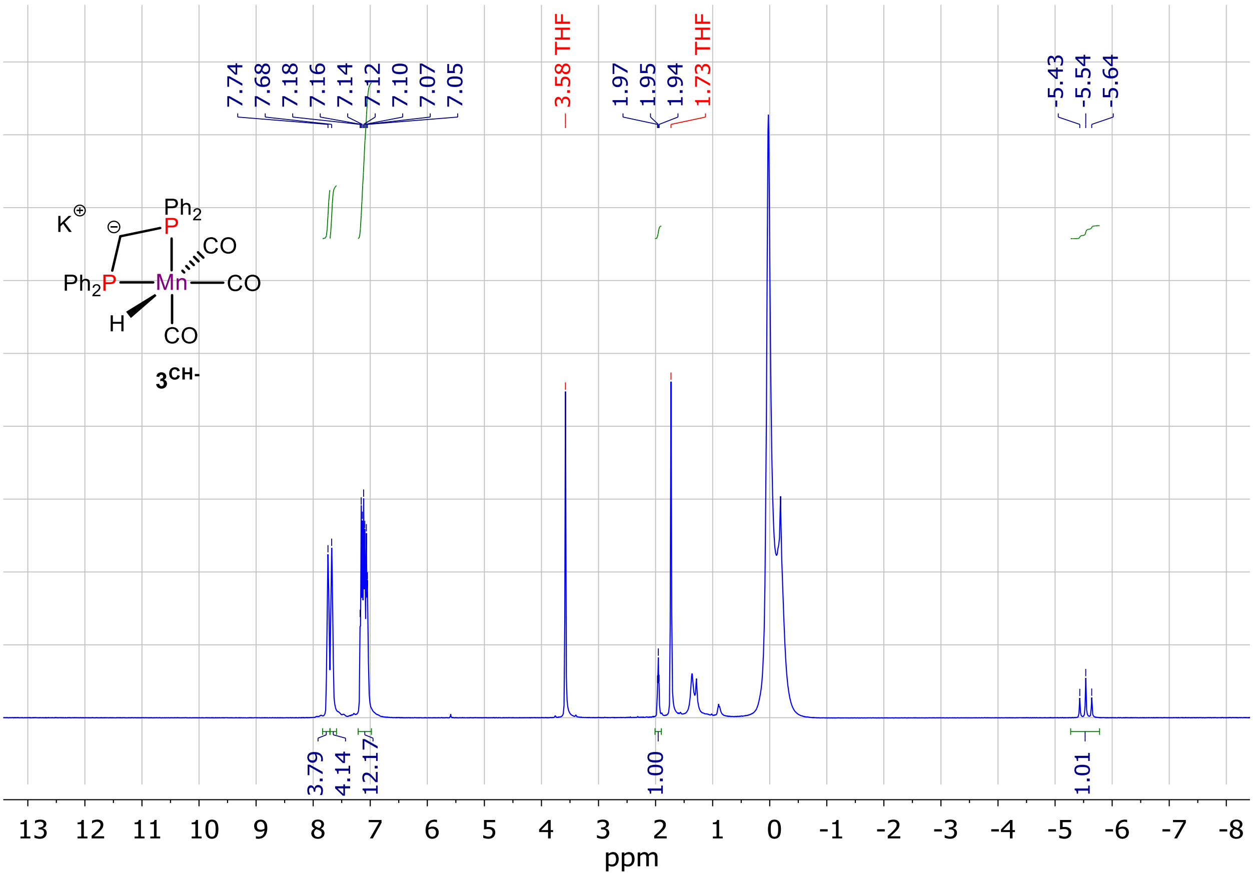

Figure S16. ^{1}H NMR spectrum of complex **3**$^{CH-}$ (400.1 MHz, THF-d_8, 243 K).

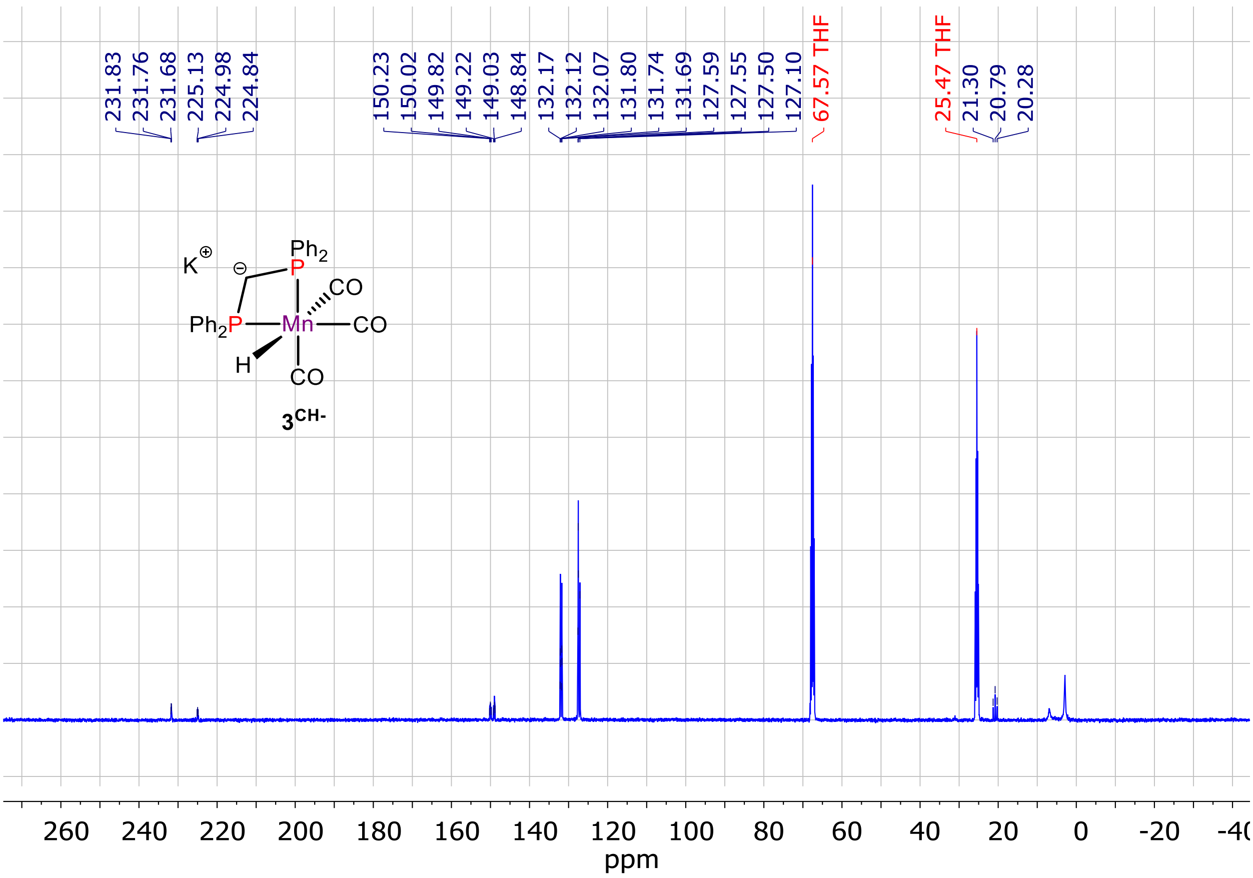

Figure S17. ^{13}C NMR spectrum of complex *fac*-**3**$^{CH-}$ (150.9 MHz, THF-d_8, 243 K).

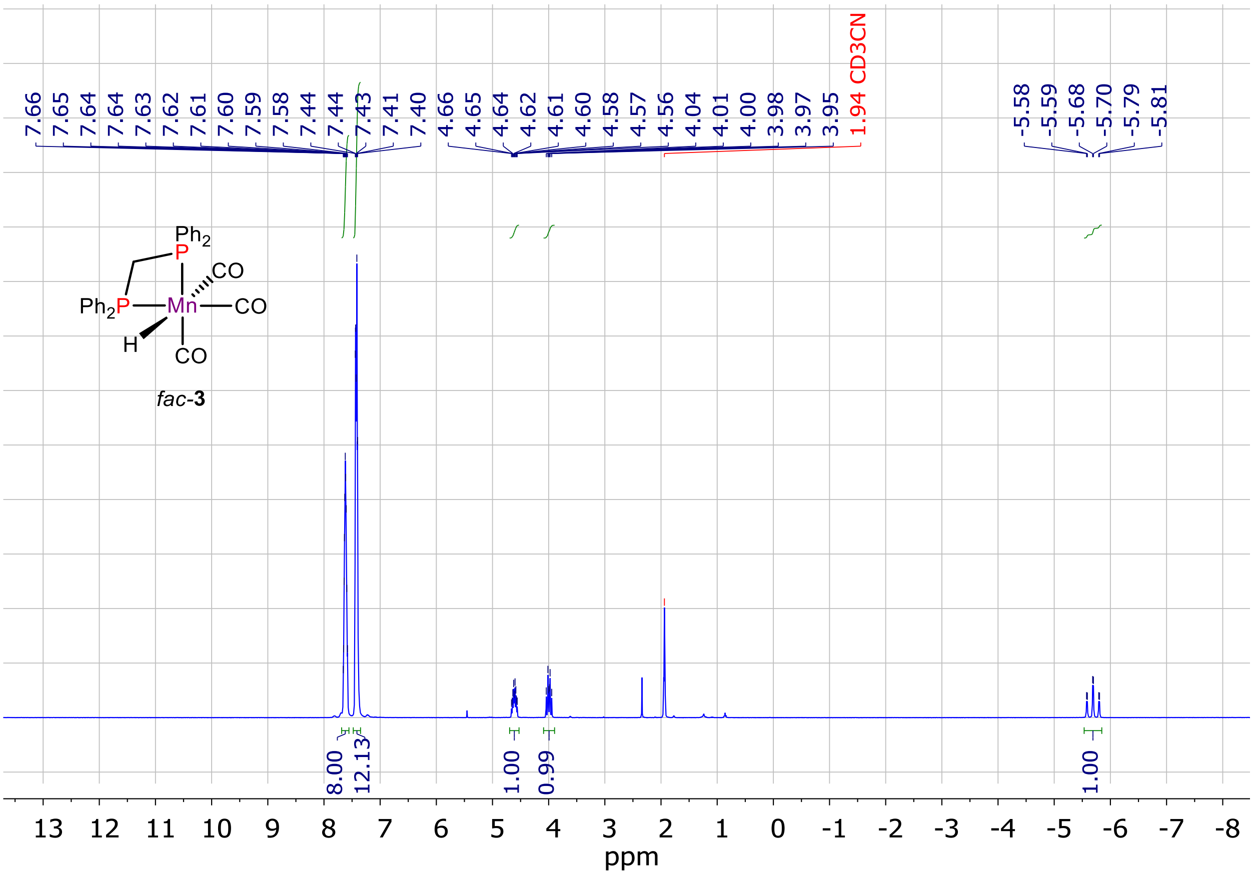

Figure S18. ^{1}H NMR spectrum of complex **3** (400.1 MHz, CD_3CN, 298 K).

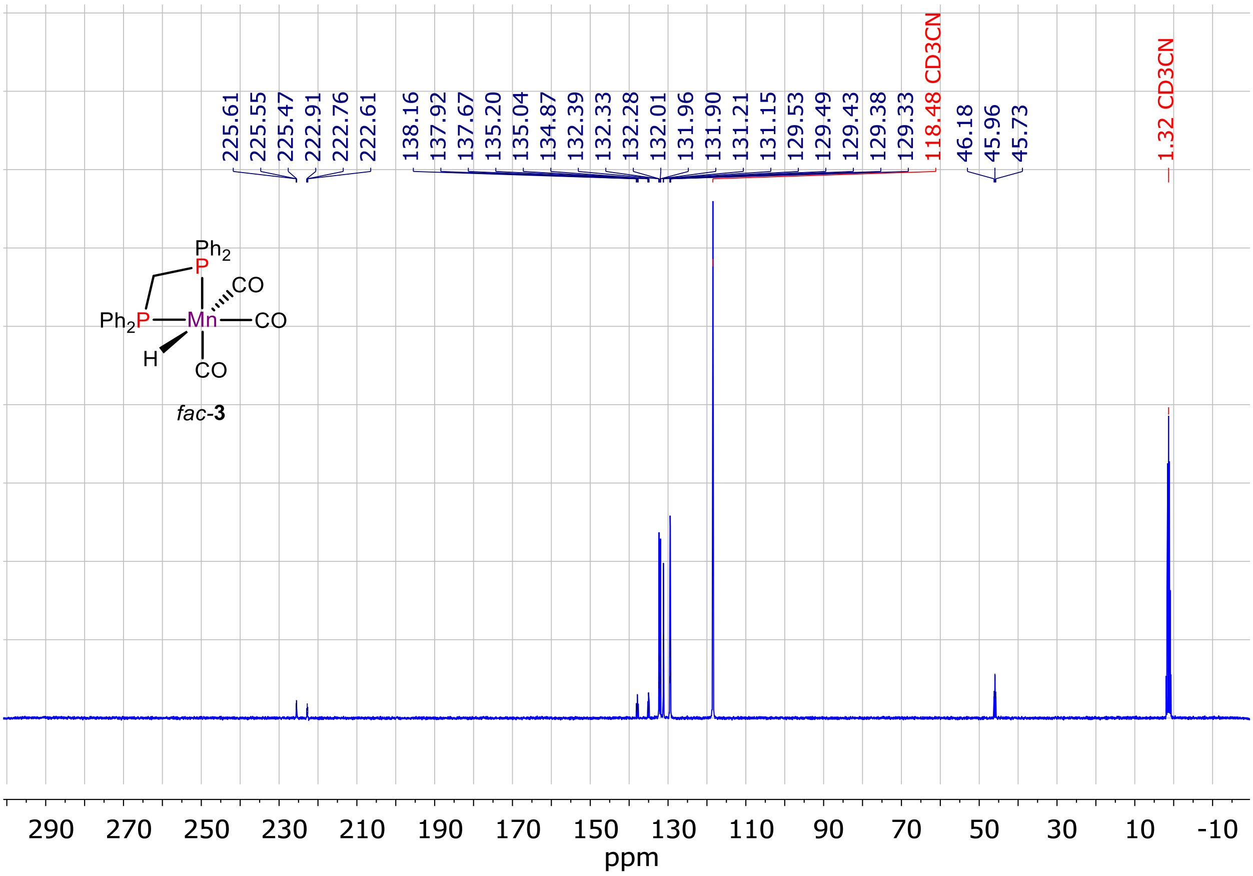

Figure S19. ^{13}C NMR spectrum of complex *fac*-**3** (150.9 MHz, CD_3CN, 298 K).

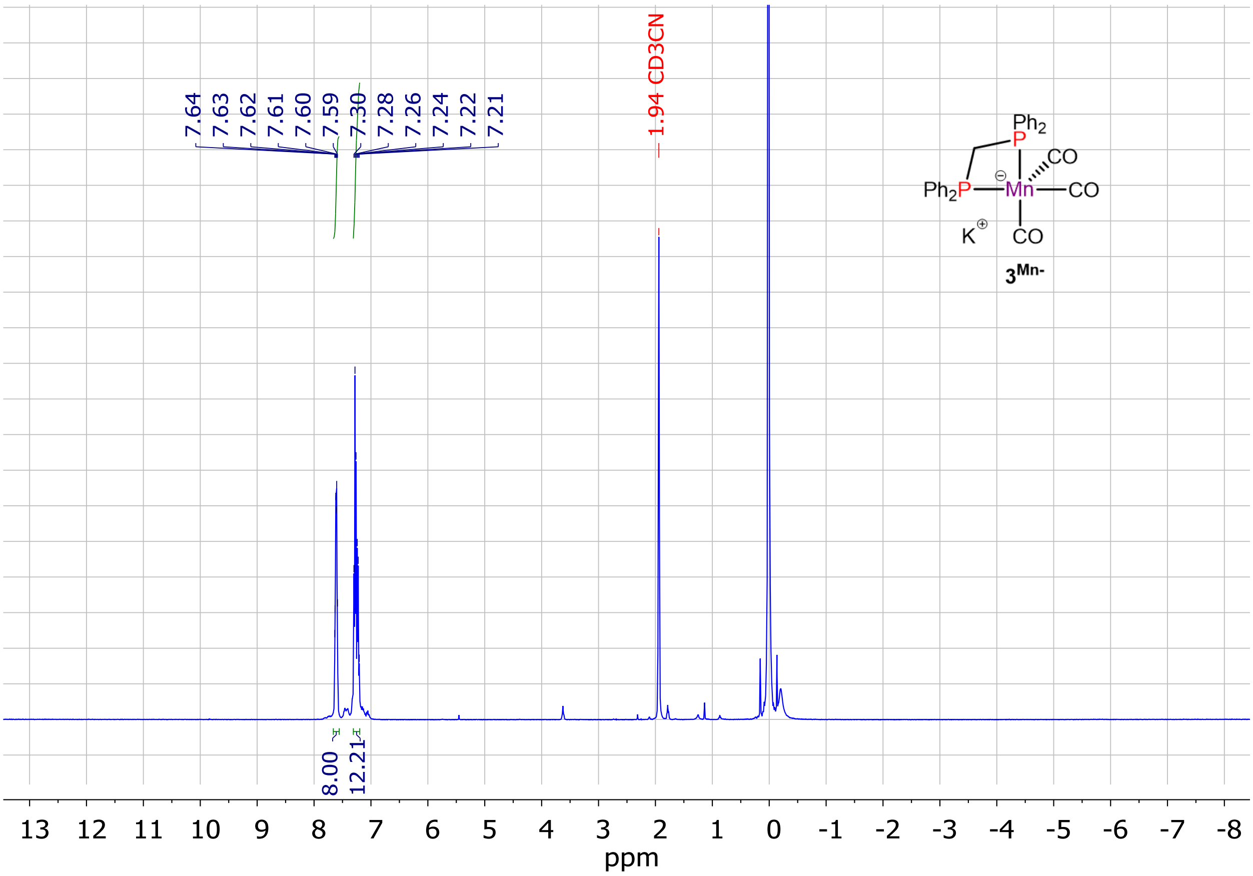

Figure S20. ^{1}H NMR spectrum of complex **3^{Mn-}** (400.1 MHz, CD_3CN, 263 K).

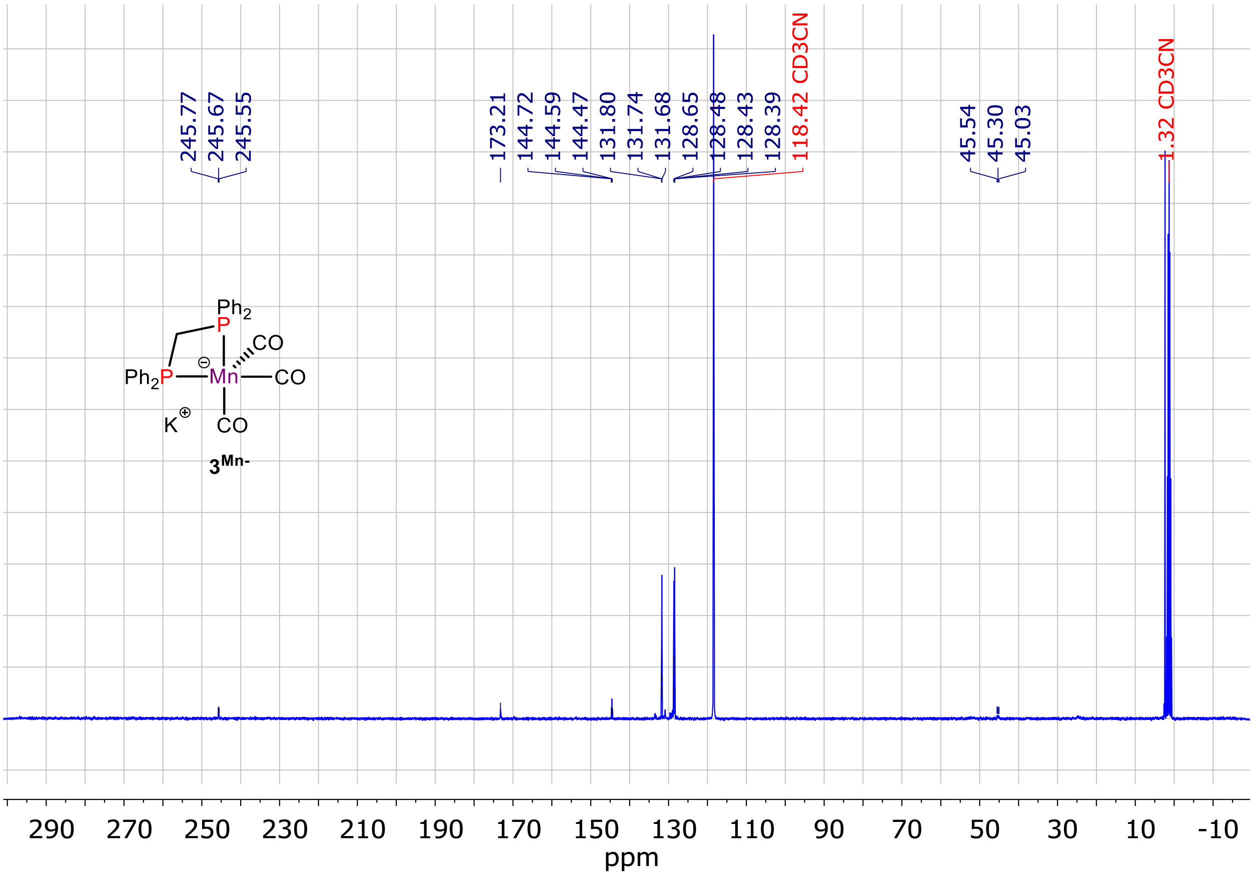

Figure S21. ^{13}C NMR spectrum of complex **3^{Mn-}** (150.9 MHz, CD_3CN, 263 K).

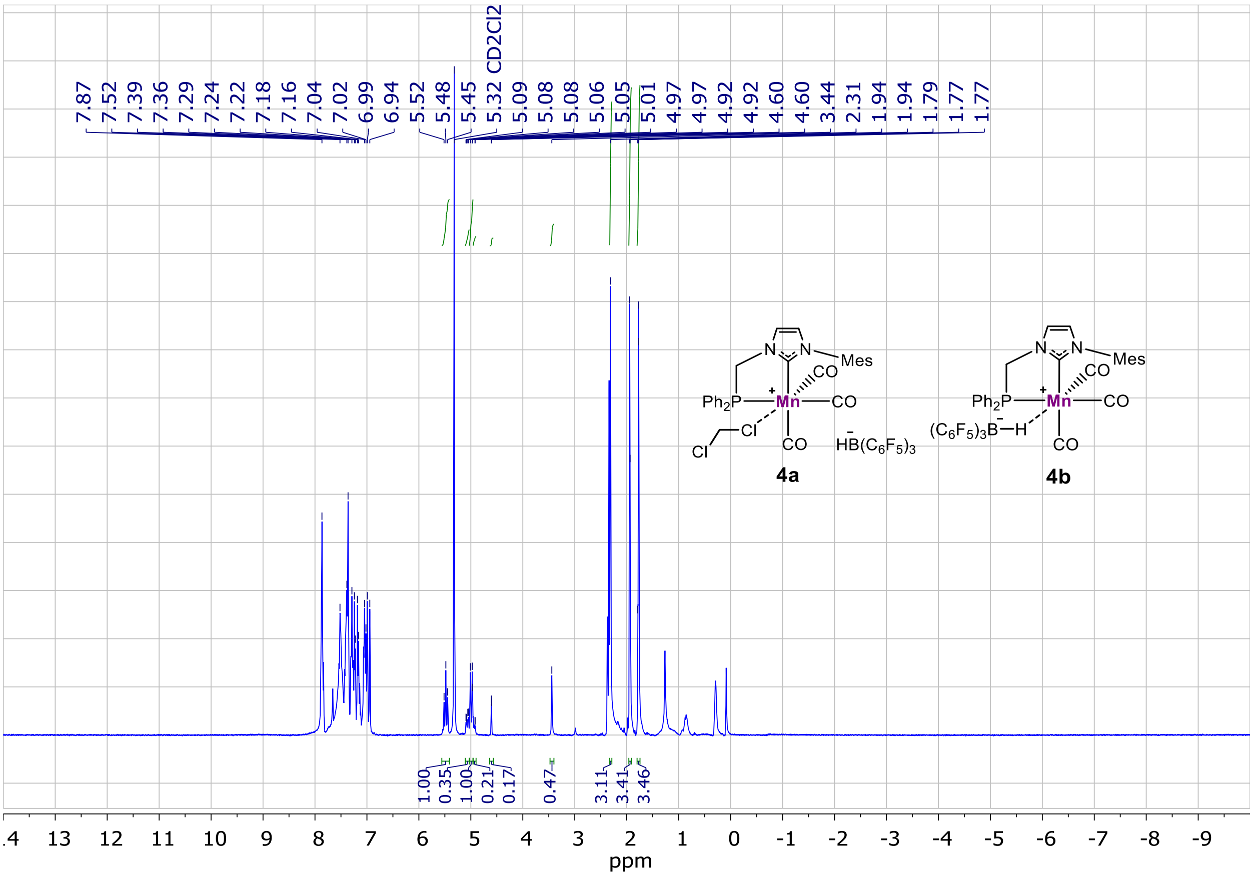

Figure S22. ^{1}H NMR spectrum of mixture *fac*-**4a** and *fac*-**4b** (400.1 MHz, CD_2Cl_2, 298 K).

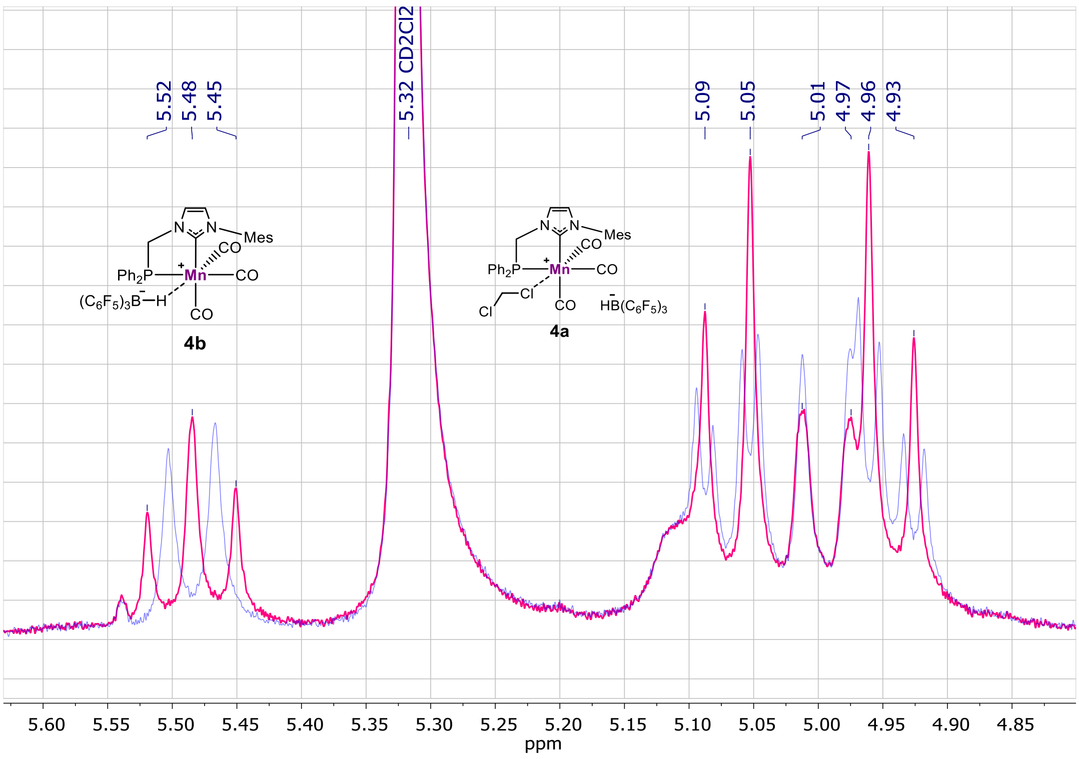

Figure S23. ^{1}H{^{31}P} NMR spectra of mixture *fac*-**4a** and *fac*-**4b** with selectinely decoupled δ_P 78.1 ppm (*blue line*) and with selectinely decoupled δ_P 71.1 ppm (*pink line*) (400.1 MHz, CD_2Cl_2, 298 K).

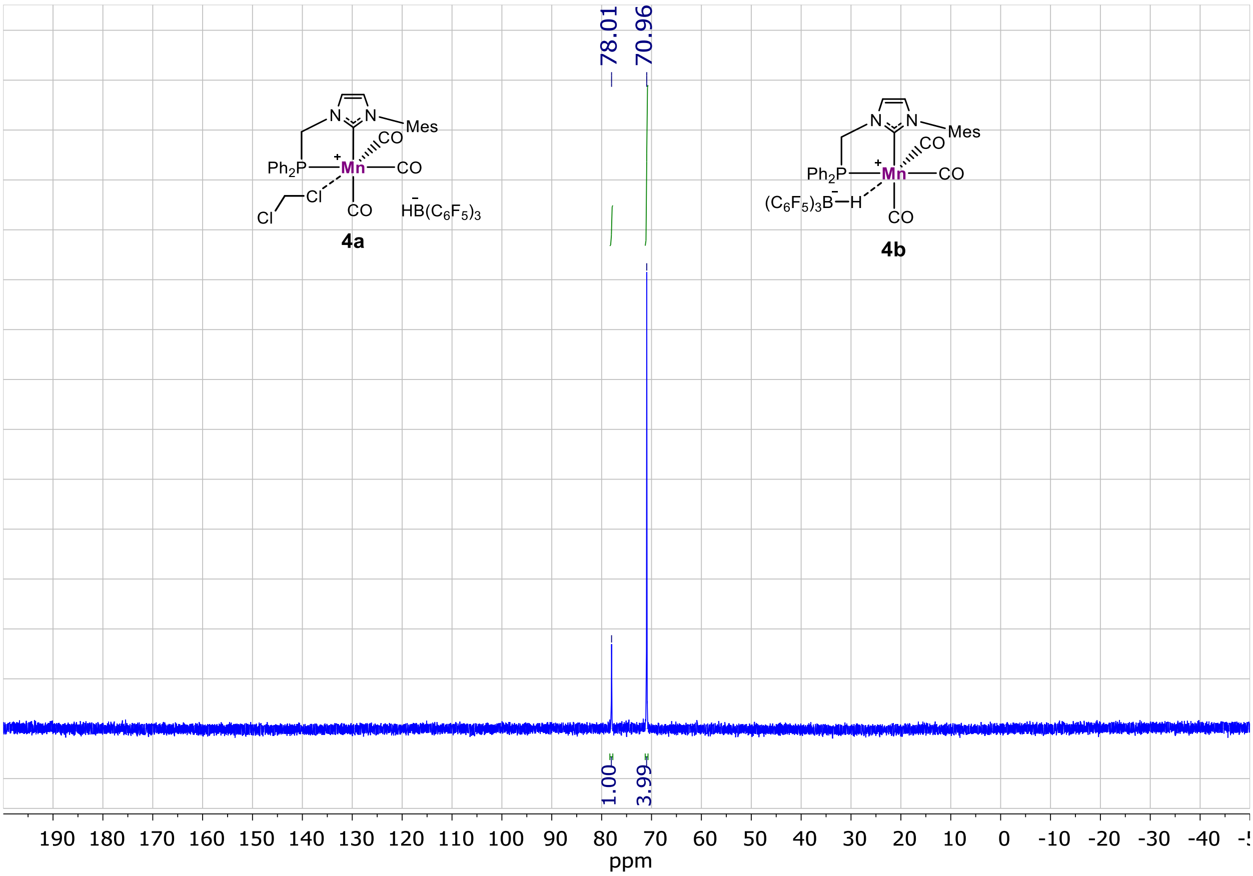

Figure S24. $^{31}P\{^{1}H\}$ NMR spectrum of mixture *fac*-**4a** and *fac*-**4b** (162.0 MHz, CD_2Cl_2, 298 K).

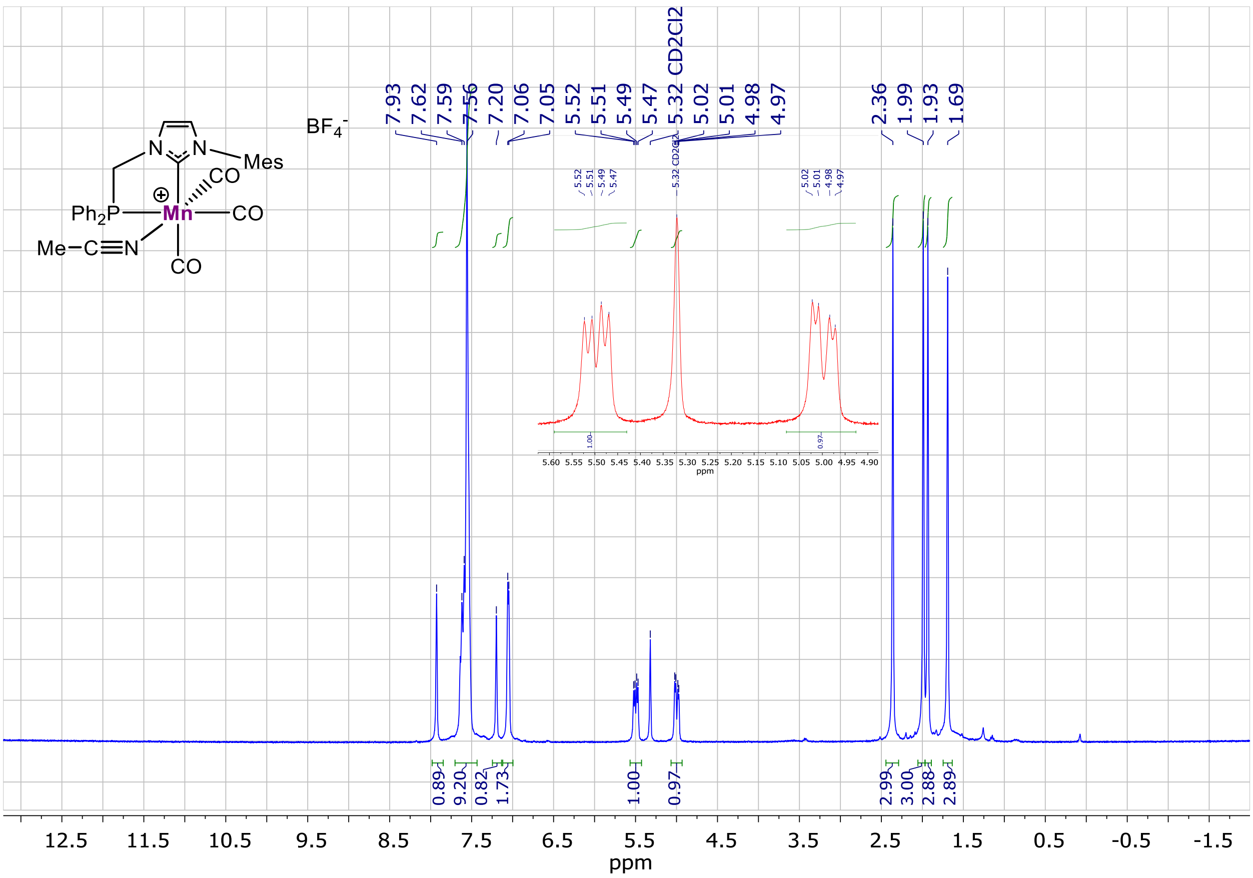

Figure S25. ^{1}H NMR spectrum of complex $\mathbf{4^{MeCN}}$ (400.1 MHz, CD_2Cl_2, 298 K).

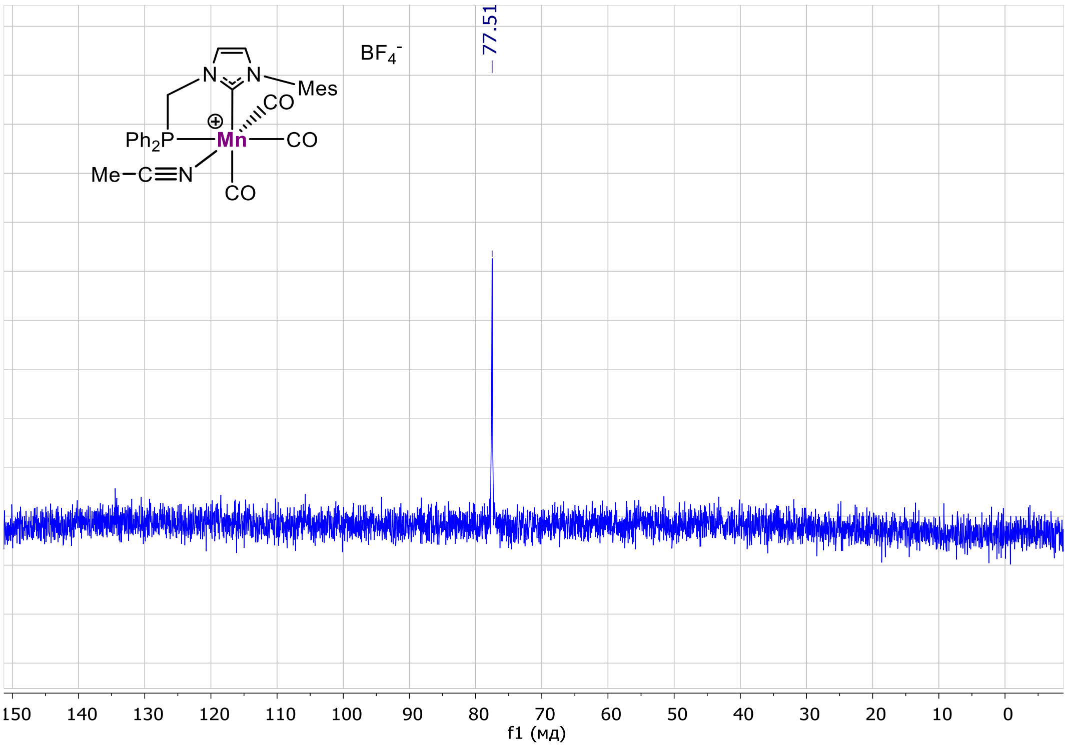

Figure S26. $^{31}P\{^{1}H\}$ NMR spectrum of complex **4^{MeCN}** (162.0 MHz, C_6D_6, 298 K).
